# Activation of human STING by a molecular glue-like compound

**DOI:** 10.1038/s41589-023-01434-y

**Published:** 2023-10-12

**Authors:** Jie Li, Stephen M. Canham, Hua Wu, Martin Henault, Lihao Chen, Guoxun Liu, Yu Chen, Gary Yu, Howard R. Miller, Viktor Hornak, Scott M. Brittain, Gregory A. Michaud, Antonin Tutter, Wendy Broom, Mary Ellen Digan, Sarah M. McWhirter, Kelsey E. Sivick, Helen T. Pham, Christine H. Chen, George S. Tria, Jeffery M. McKenna, Markus Schirle, Xiaohong Mao, Thomas B. Nicholson, Yuan Wang, Jeremy L. Jenkins, Rishi K. Jain, John A. Tallarico, Sejal J. Patel, Lianxing Zheng, Nathan T. Ross, Charles Y. Cho, Xuewu Zhang, Xiao-Chen Bai, Yan Feng

**Affiliations:** 1 Department of Biophysics, University of Texas Southwestern Medical Center, Dallas, TX USA; 2 Novartis Institutes for BioMedical Research, Cambridge, MA USA; 3 Novartis Institutes for BioMedical Research, San Diego, CA USA; 4 Aduro Biotech, Inc., Berkeley, CA USA; 5 Department of Pharmacology, University of Texas Southwestern Medical Center, Dallas, TX USA; 6 Department of Cell Biology, University of Texas Southwestern Medical Center, Dallas, TX USA

**Keywords:** High-throughput screening, Structural biology, Small molecules, Cancer therapy, Immunology

## Abstract

Stimulator of interferon genes (STING) is a dimeric transmembrane adapter protein that plays a key role in the human innate immune response to infection and has been therapeutically exploited for its antitumor activity. The activation of STING requires its high-order oligomerization, which could be induced by binding of the endogenous ligand, cGAMP, to the cytosolic ligand-binding domain. Here we report the discovery through functional screens of a class of compounds, named NVS-STGs, that activate human STING. Our cryo-EM structures show that NVS-STG2 induces the high-order oligomerization of human STING by binding to a pocket between the transmembrane domains of the neighboring STING dimers, effectively acting as a molecular glue. Our functional assays showed that NVS-STG2 could elicit potent STING-mediated immune responses in cells and antitumor activities in animal models.

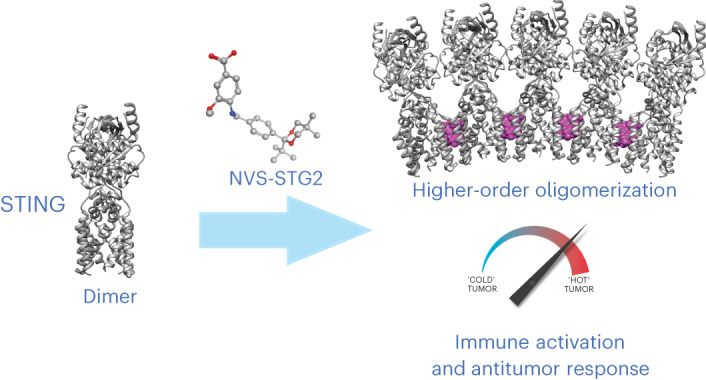

## Abstract (web summary)

A cell-based phenotypic screen led to the discovery of compounds called NVS-STGs, which bind to the N-terminal domain of STING and act as a molecular glue to induce higher-order oligomerization and activation.

## Main

The protein STING (also known as TMEM173, MITA, MPYS or ERIS) is a central node in the host cytosolic DNA surveillance pathway, which is critical for the innate immune response against pathogen infections and cancer^1,2^. Extensive studies have been devoted to understanding its regulatory mechanisms and exploiting its activity for a variety of therapeutic avenues including cancer, inflammatory diseases and pathogen infections^3–11^.

STING is a dimeric transmembrane protein residing on the endoplasmic reticulum membrane. The N-terminal transmembrane region (amino acids 1–140) of STING constitutes the transmembrane domain (TMD), which contains four transmembrane helices from each subunit organized in an intertwined manner to stabilize the dimeric state^12^. The C-terminal cytosolic region of STING forms a butterfly-shaped dimer that functions as the ligand-binding domain (LBD). STING is normally activated by the binding of cyclic di-nucleotides (CDNs) to the LBD^2^. The CDN ligands include the bacterially derived 3′–5′ phosphodiester-linked CDNs^13,14^, such as c-di-AMP, c-di-GMP and 3′,3′-cGMP-AMP (3′,3′-cGAMP), and the mammalian secondary messenger 2′,3′-cGMP-AMP (cGAMP) produced by the cytosolic DNA sensor cyclic GMP-AMP synthase (cGAS)^15,16^. Binding of CDNs to the LBD induces a notable conformational change in STING, including a 180° rotation of the LBD relative to the TMD, which enables the formation of the high-order oligomer and translocation from the endoplasmic reticulum to the Golgi^12,17–20^. Activated STING oligomers recruit and activate the downstream kinase TANK-binding kinase 1 (TBK1)^21,22^. TBK1 phosphorylates STING and the transcription factor interferon regulatory factor 3 (IRF3), which is then transported to the nucleus to activate the transcription of IFN and inflammatory cytokine genes^23^. The type I interferon (IFN-I) class of cytokines has been of interest for activating T cells and is important for an effective antitumoral response^24^. Intratumoral injections of CDN analogs have demonstrated efficacy in mouse models of cancer^25,26^. Several CDN analogs and low-molecular-weight compounds binding to the LBD of STING are currently under investigation in clinical trials^4,27^.

Here we report the discovery of a class of STING agonists that show potent antitumor activity. Our cryo-EM structures of STING bound to one of these compounds show that they activate STING through a distinct molecular glue-like mechanism.

## Results

### Identification of NVS-STG2, a small molecule STING agonist

**Fig. 1:**
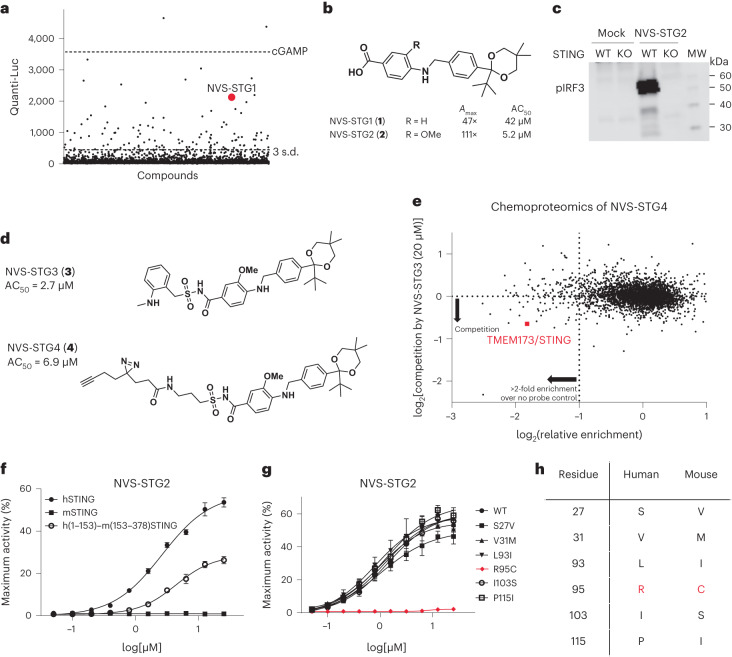
Identification of NVS-STG2 as a potent allosteric small molecule STING agonist. **a**, High-throughput screen of a 250,000 small-molecule compound library with the THP1-Dual reporter cell line identified NVS-STG1 (red circle). One representative from biological duplicates is shown. **b**, Structures of NVS-STG1 and its more potent analog NVS-STG2. AC_50_, half-maximal activity concentration; *A*_max_, maximum activity. **c**, NVS-STG2 induces STING-dependent IRF3 phosphorylation in THP1 cells. One representative from three biological replicates is shown. **d**, Structures of NVS-STG3 and PAL probe, NVS-STG4. **e**, Identification of proteins that are enriched by NVS-STG4 and where this labeling can be competed by NVS-STG3. The *x* axis denotes the enrichment observed in the presence of 1 µM NVS-STG4 relative to 0 µM NVS-STG4 (log_2_(0 µM NVS-STG4/1 µM NVS-STG4)). STING(TMEM173) was enriched over twofold with PAL probe NVS-STG4 over no probe control in chemoproteomics pull-down. Proteins below the dashed horizontal line show reduction in the enrichment in the presence of 20 µM NVS-STG3. STING(TMEM173) was also competed by adding NVS-STG3. **f**, NVS-STG2 selectively activates hSTING (solid circle), but not mSTING (solid square). A fusion STING with hSTING N-terminal TMD (1–153) and mouse C-terminal LBD (153–378) responds to NVS-STG2 (empty circle). **g**, hSTING mutants (S27V, V31M, L93I, R95C, I103S, P115I) were generated according to key amino acid differences between human and mouse STING N-terminal TMD. NVS-STG2 activity is sensitive to hSTING R95C mutation. **h**, Comparison of residues in human and mouse STING tested by mutation in **g**. In **f** and **g**, the axis represents concentrations of STG2 in log scale, and the symbols and error bars represent mean and s.e.m., respectively, from three biological replicates. KO, knockout; MW, molecular weight. Source data

To identify non-CDN small molecule activators of STING, we screened a subset of the Novartis chemical library (250,000 compounds at 50 µM) using THP1-Dual cells containing an interferon stimulated response element-luciferase (ISRE-Luc) reporter gene (Fig. 1a). Primary hits (overall hit rate of 0.25%) were confirmed in dose-response assays of up to 100 µM in the same reporter gene assay, as well as using THP1-Dual STING-knockout cells to filter out STING-independent hits. NVS-STG1 (**1**) was the sole STING-dependent hit identified in the screen (Fig. 1b).

**Extended Data Fig. 1:**
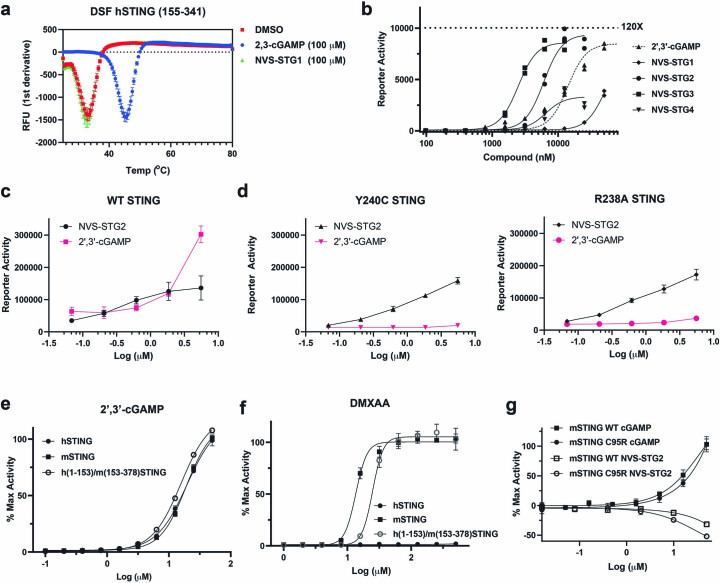
NVS-STG2 is an allosteric small molecule human STING agonist. **a**, cGAMP (blue circle, n = 14 independent samples), but not NVS-STG1 (green triangle, n = 2 independent samples), induces a dramatic (>10 °C) thermal shift in human STING C-terminal LBD (155-341) in differential scanning fluorimetry as compared to DMSO treated samples (red square, n = 14 independent samples). **b**, NVS-STG1-4 compound activity in comparison to cGAMP dose response curve in the activation of human STING in the THP1-Dual reporter cell line (n = 2 independent samples in each compound dose). **c** and **d**, STING LBD mutants Y240C (triangle) and R238A (square) lose response to cGAMP entirely but retain full response to NVS-STG2 (mean +/− SD, n = 3 independent samples in each compound dose). **e**, cGAMP activates human STING (solid circle), mouse STING (solid square), as well as a fusion STING with human STING N-terminal TMD (1-153) and mouse C-terminal LBD (153-378) (empty circle), mean +/− SD, n = 3 independent samples in each compound dose. **f**, Mouse LBD selective ligand DMXAA activates mouse STING (solid square), as well as a fusion STING with human STING N-terminal TMD (1-153) and mouse C-terminal LBD (153-378) (empty circle), but not human STING (solid circle), mean +/− SD, n = 3 independent samples in each compound dose. **g**, Mouse STING C95R mutant was not sufficient to render human STING selective NVS-STG2 active toward mouse STING (mean +/− SD, n = 3 independent samples in each compound dose). Source data

We then investigated whether NVS-STG1 bound to the STING LBD similar to the cognate substrate cGAMP. We performed differential scanning fluorimetry (DSF)^28^ with the LBD of human STING (hSTING) (aa 155–341). While treatment with cGAMP induced a marked thermal shift (>10 °C), no thermal stabilization by NVS-STG1 was observed at 100 µM (Extended Data Fig. 1a). These results suggested that NVS-STG1 does not interact with the LBD and therefore might activate STING through a distinct mechanism.

In efforts to better understand the mechanism of action of NVS-STG1, we initiated chemistry efforts to improve the potency. The initial structure activity relationship of the scaffold demonstrated the carboxylic acid to be important (esters, amides or other nonacidic functionalities were inactive). Likewise, the gem-di-methyl 1,3-dioxoane group was also important as analogs lacking the gem-di-methyl substitution or replacement of the 1,3-dioxane with a 1,3-dioxolane were inactive (AC_50_ value > 50 µM). A bulky, hydrophobic group appended to the 1,3-dioxane was needed as smaller substituents than the *t*-Bu group abolished activity. NVS-STG2 (**2**), a compound containing a 3-methoxy modification of the 4-aminobenzoic group of NVS-STG1, showed over 100-fold induction of ISRE-Luc reporter and almost tenfold improvement in the AC_50_ value compared with NVS-STG1 (Fig. 1b and Extended Data Fig. 1b). Consistently, NVS-STG2 strongly induced phosphorylation of IRF3, the key transcription factor downstream of the STING pathway, in a strictly STING-dependent manner (Fig. 1c).

Active compounds identified in phenotypic cell-based pathway screens can act either directly or indirectly on the desired target. To build confidence that NVS-STG1/2 acts directly on STING, we took a photoaffinity labeling (PAL) and quantitative chemical proteomics approach^29^. The carboxylic acid on NVS-STG2 could be replaced with an *N*-acyl sulfonamide, a carboxylic acid isostere, as shown by NVS-STG3 (**3**, THP1-Dual ISRE-Luc Assay, AC_50_ = 2.7 µM; Fig. 1d and Extended Data Fig. 1b), and retained good potency. A photoactivatable diazirine cross-linker and an alkyne moiety for click-chemistry-mediated affinity capture^30,31^ was appended to the *N*-acyl sulfonamide to generate the PAL probe, NVS-STG4 (**4**, THP1-Dual ISRE-Luc Assay, AC_50_ of 6.9 µM; Fig. 1d and Extended Data Fig. 1b). THP1 cells were incubated with varying concentrations of NVS-STG4 to identify proteins with saturable binding. NVS-STG3 was used as a free competitor, due to its potency and structural similarity to NVS-STG4. The cell lysates were analyzed by quantitative mass spectrometry, which identified STING as one of the limited number of proteins enriched by NVS-STG4 (Fig. 1e). This labeling was competed by NVS-STG3, suggesting it is likely that the NVS-STG series acts directly on hSTING.

### NVS-STG2 targets the STING TMD

The combined results vide supra suggested that NVS-STGs directly interact with hSTING, but likely not through the LBD. To strengthen the observation, we mutated key LBD residues in hSTING, Y240C and R238A, which are known to abrogate CDN binding^32^. Our results showed that NVS-STG2 maintained full agonist activity towards the Y240C and R238A mutants, whereas cGAMP was completely inactive (Extended Data Fig. 1c,d).

Lack of binding of NVS-STG1/2 toward the LBD of hSTING suggested that the compounds may modulate STING through the N-terminal TMD. We have shown recently that C53, a hydrophobic compound, could bind a cryptic pocket in the human TMD and induce its activation^33^. To demonstrate the involvement of the TMD in NVS-STG1/2-dependent STING activation, we performed a series of mutagenesis studies. STING activation by small molecule ligands is known to have notable species differences. A well-known cancer drug candidate, DMXAA, is a mouse-specific STING agonist^34,35^. Notably, NVS-STG2 specifically activated hSTING, but not mouse STING (mSTING) (Fig. 1f). We took advantage of the species selectivity of NVS-STG2 and made an hSTING(1–153)–mSTING(153–378) chimera that contained the hSTING TMD and mSTING LBD. This chimera responded fully to cGAMP and DMXAA (Extended Data Fig. 1e,f), consistent with the fact that both ligands can act on the LBD of mSTING(153–378). NVS-STG2, which does not activate mSTING, induced substantial activation of the hSTING TMD–mSTING LBD chimera (Fig. 1f), suggesting that NVS-STG2 activates hSTING by binding to the TMD.

To understand the NVS-STG2-binding site better, we chose a series of residues with greatest transmembrane topology differences predicted by a hidden Markov model^36,37^ and generated specific hSTING-to-mSTING TMD point mutants (S27V, V31M, L93I, R95C, I103S, P115I) to determine whether these mutations would modulate STING activation by NVS-STG2. While all point mutations retained full capacity to be activated by cGAMP, the R95C mutant lost all activity induced by NVS-STG2 (Fig. 1g,h), suggesting a potential key role of R95 in NVS-STG2 binding, and further supporting NVS-STG2 as an N-terminal allosteric agonist for hSTING. Disappointingly, the mSTING C95R was unable to restore the activity of NVS-STG2 against mSTING, suggesting that other proximal residues in the pocket of NVS-STG2 must also be of importance (Extended Data Fig. 1g).

### Structures of the STING oligomer bound to NVS-STG2

**Fig. 2:**
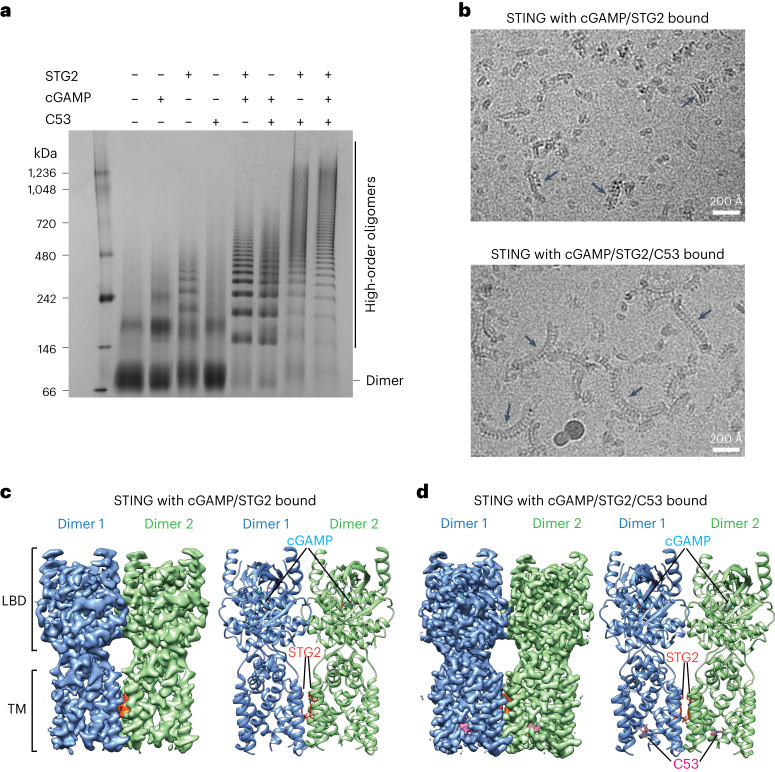
Cryo-EM structures of hSTING bound to NVS-STG2. **a**, Induction of STING oligomerization by cGAMP, C53, NVS-STG2 and their different combinations. STG2, NVS-STG2. One representative gel from three replicates is shown. **b**, Micrographs of STING bound to cGAMP/NVS-STG2 or cGAMP/NVS-STG2/C53. Images shown are representatives from *n* = 3,352 and *n* = 4,921 images for the cGAMP/NVS-STG2 and cGAMP/NVS-STG2/C53 samples, respectively. Arrows highlight STING oligomers. **c**, Cryo-EM density map and atomic model of STING bound to cGAMP/NVS-STG2. **d**, Cryo-EM density map and atomic model of STING bound to cGAMP/NVS-STG2/C53. TM, transmembrane. Source data

We have shown previously that activation of STING by ligands can be assayed in vitro by monitoring the high-order oligomerization of purified STING with native gels^33^. We tested whether NVS-STG2 could induce the oligomerization of hSTING using this assay. Consistent with our previous results, STING in the absence of any ligand existed predominately in the dimeric state, while cGAMP and C53 individually induced the formation of the tetramer and relatively small high-order oligomers (Fig. 2a). The presence of both cGAMP and C53 led to more robust high-order oligomerization. Notably, NVS-STG2 alone was able to induce high-order oligomers of STING. The combinations of NVS-STG2 with cGAMP, C53 or both promoted the formation of more and larger oligomers of STING. These results suggested that NVS-STG2 induces the oligomerization and activation of hSTING by binding to a site in the TMD different from that targeted by C53.

**Extended Data Fig. 2:**
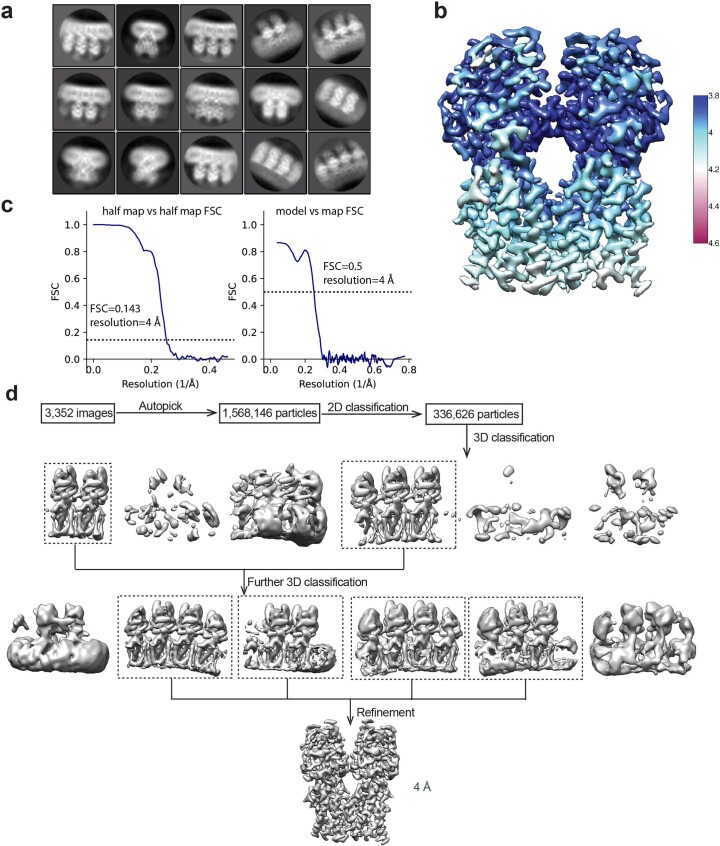
Image processing procedure of STING bound to cGAMP/NVS-STG2. **a**, 2D class averages of segmented high-order oligomers of STING. **b**, Local resolution map of the final 3D reconstruction. **c**, Gold-standard FSC curve of the final 3D reconstruction (left) and FSC between the map and atomic model (right). **d**, Image processing procedure.

**Extended Data Fig. 3:**
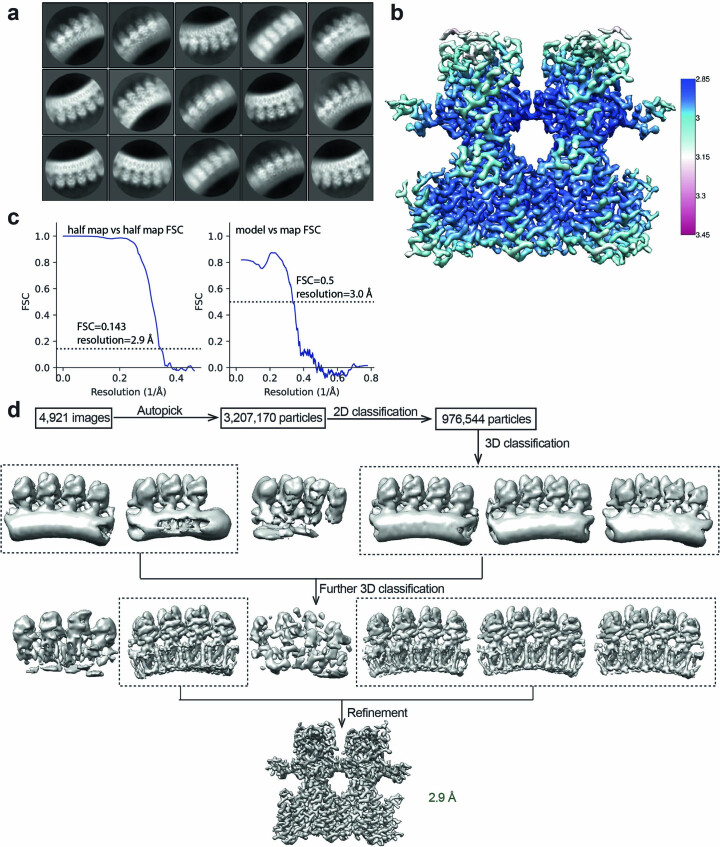
Image processing procedure of STING bound to cGAMP/NVS-STG2/C53. **a**, 2D class averages of segmented high-order oligomers of STING. **b**, Local resolution map of the final 3D reconstruction. **c**, Gold-standard FSC curve of the final 3D reconstruction (left) and FSC between the map and atomic model (right). **d**, Image processing procedure.

**Extended Data Fig. 4:**
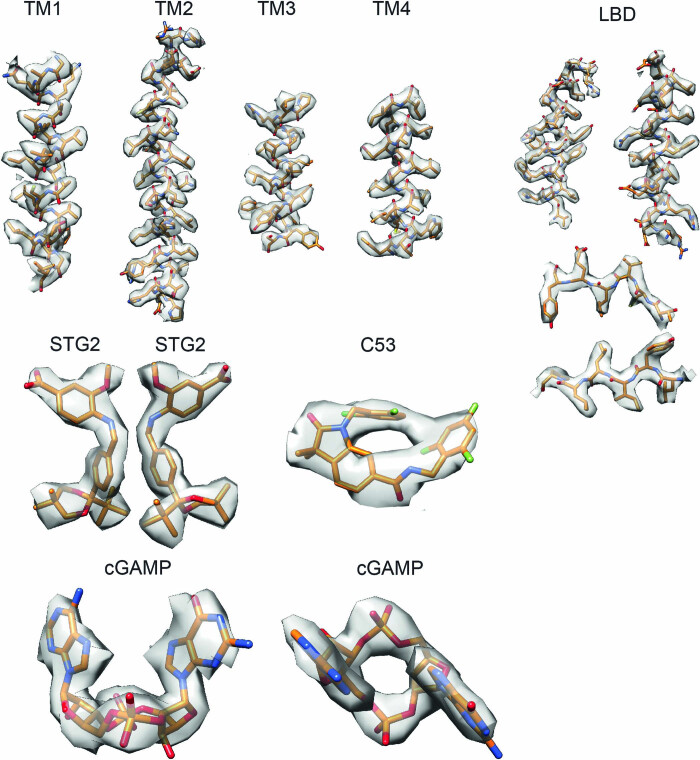
Sample density maps of various parts of the structure. Various parts of the structure of STING bound to cGAMP/NVS-STG2/C53 are show as sticks with the cryo-EM density superimposed.

To understand how NVS-STG2 binds hSTING and induces its high-order oligomerization, we sought to determine the structure of STING in complex with both cGAMP and NVS-STG2 using single particle cryo-EM. Cryo-EM micrographs of STING with both cGAMP and NVS-STG2 bound showed that the protein formed oligomers consisting of approximately 3–6 dimers (Fig. 2b). As described previously, we treated the oligomers formed by the STING dimers packing in a roughly linear fashion as tetrameric units for three-dimensional (3D) reconstruction^33^. The resulting structure of the cGAMP/NVS-STG2-bound STING tetramer reached overall 4-Å resolution (Fig. 2c and Extended Data Fig. 2). Notably, strong cryo-EM densities at the TMD–TMD interface between the two STING dimers could be attributed to NVS-STG2. Nevertheless, the medium resolution of the cryo-EM map did not allow us to model NVS-STG2 unambiguously. Considering the native gel results, we reasoned that the presence of all three ligands could enhance the stability of the high-order oligomers of STING and lead to a structure of higher resolution. Indeed, cryo-EM images showed that STING bound to cGAMP/C53/NVS-STG2 formed more abundant oligomers, which were much longer (consisting of 10–30 dimers) than those induced by either cGAMP/C53 or cGAMP/NVS-STG2 (Fig. 2b)^33^. The cryo-EM map of the STING tetramer in complex with cGAMP/C53/NVS-STG2 was resolved at 2.9-Å resolution (Fig. 2d and Extended Data Fig. 3). The map shows well-defined densities for all three ligands, allowing the modeling of NVS-STG2 unequivocally (Extended Data Fig. 4). While two NVS-STG2 molecules bind to the cavity formed between the TMDs of the two neighboring STING dimers, cGAMP and C53 bind the LBD and the TMD pockets, respectively, within each STING dimer as expected.

**Extended Data Fig. 5:**
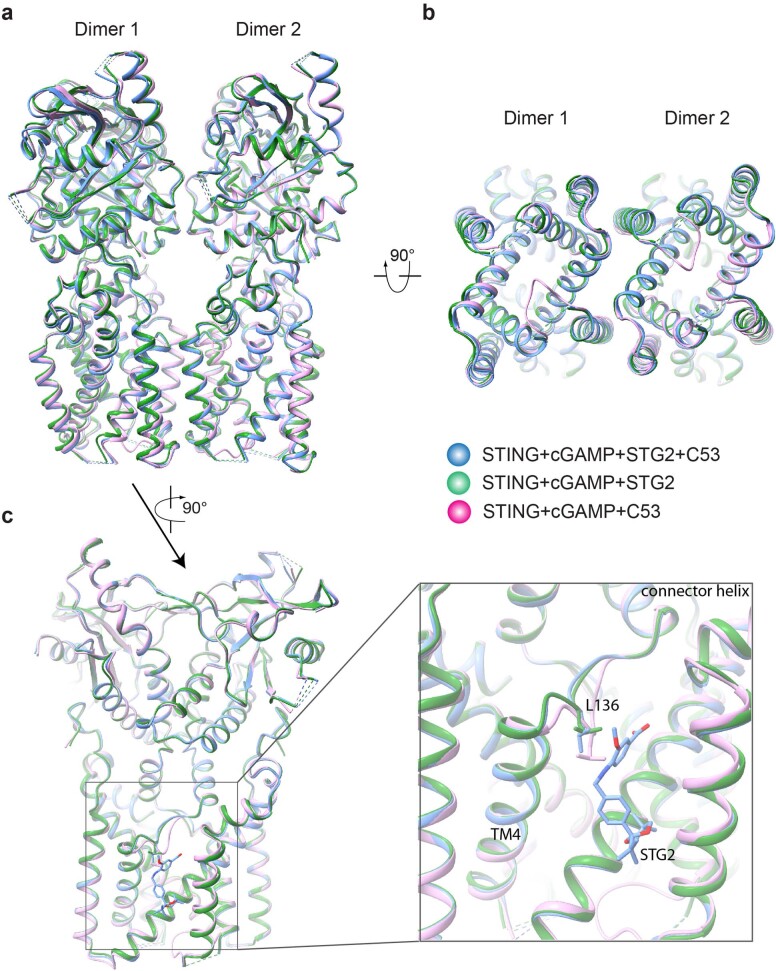
Comparison of STING bound to different ligands. **a** and **b**, Superimposition of the STING tetramer bound to cGAMP/NVS-STG2/C53, cGAMP/STG2 or cGAMP/C53 (PDB ID: 7SII). The three structures have high similarity to one another. **c**, NVS-STG2 induces a small conformational change around L136.

The tetrameric models of hSTING bound to cGAMP/NVS-STG2 or cGAMP/C53/NVS-STG2 are essentially identical to each other and to the previous structure of STING bound to cGAMP/C53 (ref. ^33^), with the pairwise root mean squared deviations among the three structures of approximately 0.45 Å (Extended Data Fig. 5a). The only notable difference between the two structures solved here and the previous cGAMP/C53-bound structure is at L136 following TM4, which in the NVS-STG2-containing structures undergoes a ~2.5-Å shift to accommodate NVS-STG2 (Extended Data Fig. 5b,c). C53 binds a cryptic pocket formed by the TMDs of the two subunits in the hSTING dimer and induces outward movements of the transmembrane helices, allowing them to engage in the TMD–TMD interaction between neighboring dimers that contributes to the high-order oligomerization^33^. The fact that the structure of STING bound to cGAMP/NVS-STG2 is nearly identical to the cGAMP/C53-bound structure shows that NVS-STG2 is able to induce the same conformational change to the STING TMD as C53, without directly engaging the C53-binding pocket. These observations further support the notion that these conformational changes in the TMD are integral parts of the oligomerization and activation mechanism of STING, rather than merely the result of binding of an artificial ligand^33^.

### NVS-STG2 as a molecular glue promotes STING oligomerization

**Fig. 3:**
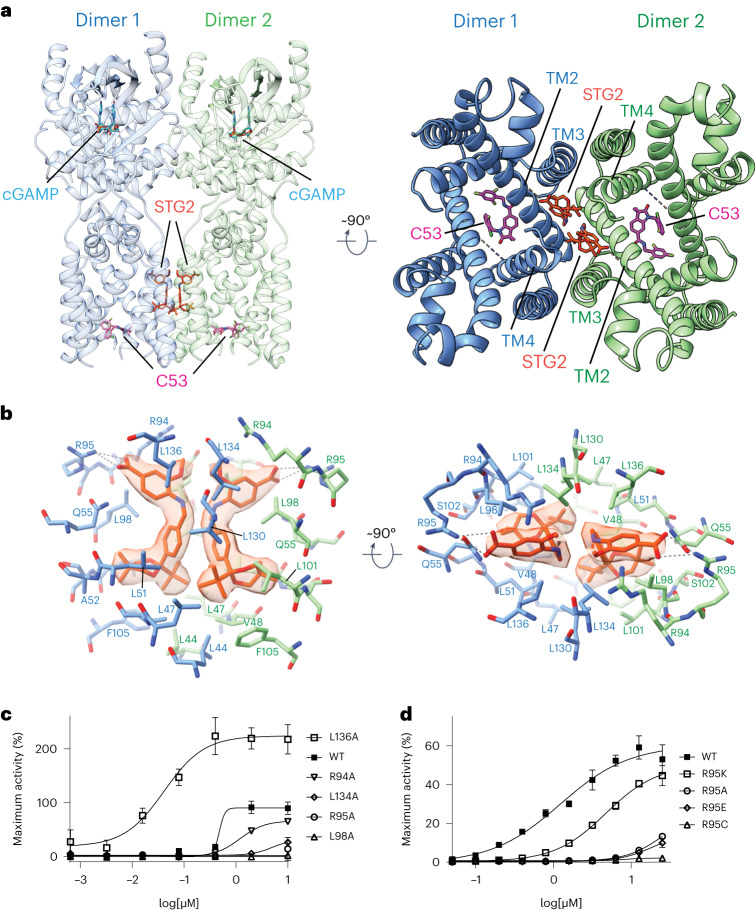
Details of the binding mode of NVS-STG2. **a**, Overviews of the binding modes of NVS-STG2 (STG2) and C53. **b**, Detailed views of the NVS-STG2-binding site. **c**, Reponses of hSTING to NVS-STG2, with key binding pocket mutations. The *x* axis represents the concentration of NVS-STG2 in log scale. **d**, Activation of hSTING R95 mutants by STG2. In **c** and **d**, data are mean and s.e.m. from three biological replicates. Source data

A molecular glue is defined as a compound that should: (1) enhance the interface of two proteins to enhance the affinity of the two proteins as either a heteromeric or homomeric complex; and (2) engage in interactions with both protein surfaces^38–40^. In both structures of STING in complex with either cGAMP/NVS-STG2 or cGAMP/C53/NVS-STG2, two NVS-STG2 molecules, packing side-by-side mainly through the central phenyl group, occupy a cavity between the TMDs of the two neighboring STING dimers (Fig. 3a). Each NVS-STG2 predominantly interacts with TM2, TM3 and TM4 of one STING dimer, and meanwhile contacts TM2 and TM4 of the adjacent STING dimer. With such a binding mode, the NVS-STG2 dimer effectively acts as a molecular glue that enhances the interface of the two STING dimers, thereby promoting the high-order oligomerization of STING.

**Extended Data Fig. 6:**
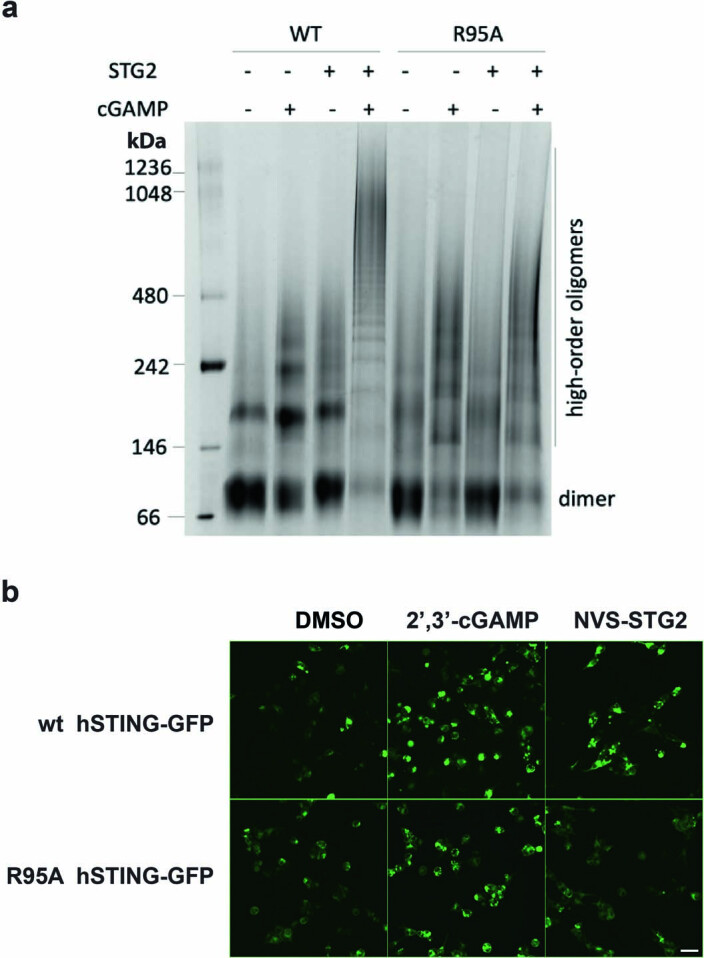
STG2-induced oligomerization of STING is dependent on R95. **a**, Induction of oligomerization of wild-type (WT) STING and the R95A mutant by cGAMP, NVS-STG2 or their combination. STG2, NVS-STG2. **b**, Fluorescent puncta-like structure formation of wild-type STING and the R95A mutant upon activation by cGAMP or NVS-STG2 (scale bar 20 µm). Data shown are representatives of three biological replicates. Source data

The cryo-EM structure confirmed that the carboxylic acid of NVS-STG2 forms a salt bridge with R95 located at the cytosolic end of TM3 in STING (Fig. 3b). R94 in STING also forms electrostatic interactions with the carboxylic acid in NVS-STG2. These interactions explain the significant role of the acidic group in NVS-STG2 for activating STING. To confirm the importance of the R95 residue for NVS-STG2 binding and activation, an R95A mutant was generated and assayed for in vitro higher-order oligomerization by native gel. The R95A mutant showed similar formation of oligomers to the wild-type protein when treated with cGAMP. However, there was a notable defect in higher-order oligomer formation when treated with NVS-STG2 alone or in combination with cGAMP (Extended Data Fig. 6a). Activation of STING triggers translocation from the endoplasmic reticulum to post-Golgi compartments in which it forms puncta-like structures which are indicative of oligomer formation^12,18,22^. To test the role of the R95A mutation in the cellular activation of STING, the wild-type or the R95A mutant with a C-terminal GFP was stably expressed in HEK293T cells. Both wild-type STING and R95A-STING were able to form bright puncta-like structures when stimulated with cGAMP. However, only the wild-type was responsive to NVS-STG2 whereas the R95A mutant had lost the ability to form puncta-like structures when stimulated with NVS-STG2 (Extended Data Fig. 6b).

Additional residues contributing to the interaction with one end of NVS-STG2 include Q55, L98, L134 and L136 of one STING dimer and L134 of the second dimer. The other end of NVS-STG2 is tightly embraced by hydrophobic residues in the TMD of STING. The 5,5-dimethyl-1,3-dioxane group plus the linked tert-butyl contacts V48, L51, A52, L101 and F105 from one STING dimer and L44, L47, L51 and L130 from the second dimer. In addition to STG2-mediated inter-dimer interactions, the TMDs of the two STING dimers make direct contacts to further stabilize the high-order oligomerization. These interactions are essentially identical to those in the previous structure of STING bound to cGAMP/C53 and have been shown by mutational analyses to be important for the oligomerization and activation of STING^33^.

To further validate the binding mode of NVS-STG2 shown in the structure, we tested additional mutations in the TMD of STING, including R94A, R95A, L98A and L134A. These mutants exhibited various degrees of reduction in NVS-STG2-simulated activation in cells using an IRSE-Luc reporter assay in transiently transfected HEK293T cells, while their responses to cGAMP were largely intact (Fig. 3c). We further examined the effects of mutating R95 on NVS-STG2-stimulated STING signaling. The results showed that the R95K mutant was partially active, whereas R95A, R95E and R95C were essentially irresponsive to NVS-STG2 (Fig. 3d)^41^. These results are consistent with the structure showing the critical role of the electrostatic interactions in the binding of NVS-STG2. Additionally, we observed an enhancement of activity in the L136A mutation. As mentioned above, binding of NVS-STG2 requires a shift of the loop containing L136 to avoid clashes (Extended Data Fig. 5c). The smaller sidechain of alanine at position 136 likely reduces the required shift and the associated energetic penalty, which underlies the enhanced activation of the L136A mutant by NVS-STG2 (Fig. 3c).

The structures provide an explanation for why NVS-STG2 can activate human but not mouse STING. Most importantly, R94 and R95 in hSTING are replaced by a histidine and cysteine, respectively, in mSTING, which lack the ability to form electrostatic interactions with the carboxylic acid in NVS-STG2. In addition, V48, Q55 and L98 in hSTING are changed to alanine, glutamate and methionine, respectively, in mSTING. These additional changes in the binding interface likely underlie the result that the C95R mutation of mSTING was unable to restore the activity of NVS-STG2 agonist mSTING (Extended Data Fig. 1g).

### In vivo antitumor activity of NVS-STG2

**Extended Data Fig. 7:**
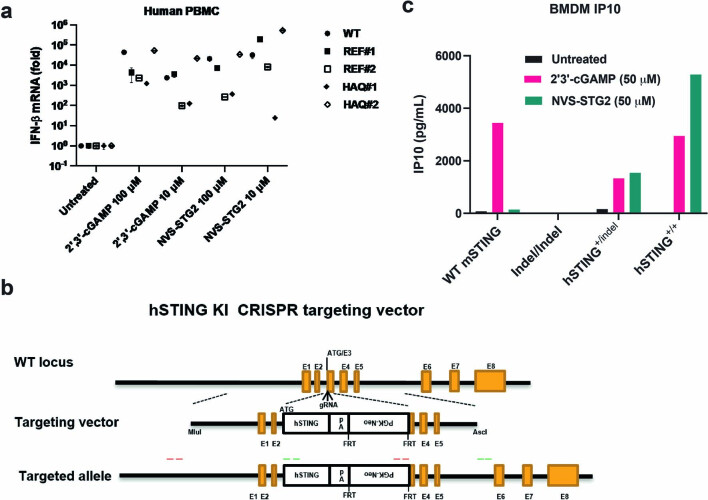
Characterization of NVS-STG2 in human PBMC and human STING KI mice BMDM. **a**, NVS-STG2 induces high level of IFN-b in all major human PBMC haplotypes (WT, REF and HAQ), in comparable level to endogenous ligand cGAMP (mean fold changes over untreated sample +/− SEM, n = 3 independent samples for each condition). **b**, Diagram of human STING CRISPR KI. **c**, Bone marrow derived macrophages (BMDM) from hSTING knock-in (KI) mice (mSTING^wt/wt^, mSTING^indel/indel^, hSTING^+/indel^, hSTING^+/+^) were stimulated with cGAMP (green) or NVS-STG2(red) and IP10 levels (n = 1 for each condition) were measured. cGAMP induces IP10 in both mouse and human STING mice BMDM, while NVS-STG2 only induces IP10 in mice with hSTING KI alleles. Source data

We further tested whether activation of STING by NVS-STG2 gave similar immune responses in relevant cellular and in vivo models. First, human peripheral blood mononuclear cells (PBMCs) from a panel of donors harboring different STING alleles (hSTING^WT/WT^, hSTING^REF/REF^, hSTING^HAQ/HAQ^) were treated with NVS-STG2 and cGAMP and induction of IFNβ was measured by quantitative PCR with reverse transcription (RT–qPCR). NVS-STG2 showed high levels of IFNβ induction comparable to cGAMP (Extended Data Fig. 7a).

The strong immune phenotype induced by NVS-STG2 suggested that NVS-STG2 might also enable the immune system to launch an in vivo antitumor response similar to CDN analogs currently undergoing clinical trials^42^. Unfortunately, the human species-specific nature of NVS-STG2 made its evaluation in standard preclinical murine tumor models challenging. To overcome this issue, CRISPR–Cas9 gene editing was leveraged to generate a hSTING knock-in mouse line ^43,44^ (Extended Data Fig. 7b). To validate the immune response in these hSTING knock-in mice, IFN responses (IP10 induction) were measured from bone marrow-derived macrophages (BMDMs). Treatment of the BMDMs isolated from the hSTING knock-in mice with NVS-STG2 induced similar level of IP10 as treatment of BMDM from either hSTING knock-in or mSTING wild-type mice with cGAMP (Extended Data Fig. 7c).

**Fig. 4:**
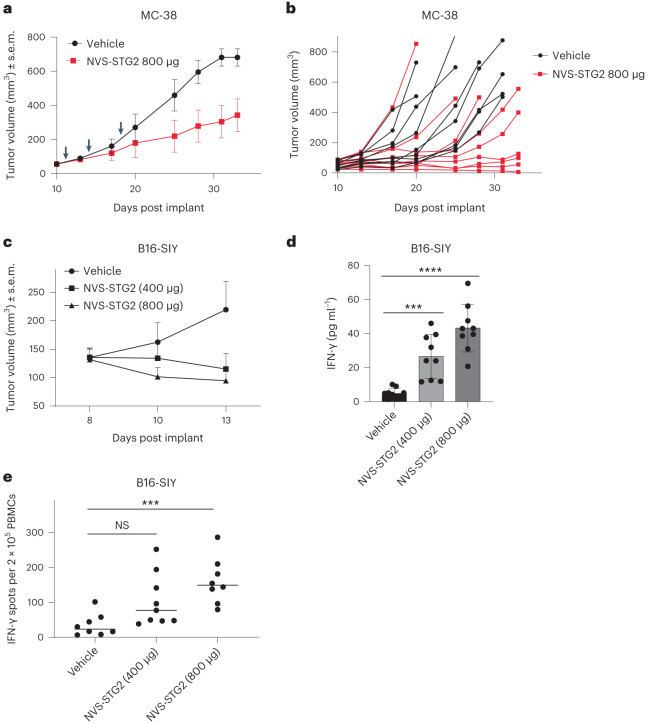
NVS-STG2 induces antitumor activity and immune response in hSTING knock-in mice. **a**, NVS-STG2 (red squares) or vehicle (black circles) was injected intratumorally to MC38 flank tumors on days 11, 14 and 18, and tumor size was measured by caliper. Data are mean ± s.e.m. (*n* = 9 mice in each group). **b**, Individual tumor growth is shown. Four of nine mice treated with NVS-STG2 (red squares) saw no tumor growth during the 33-day experimental period. **c**, NVS-STG2 at 400 μg (squares), 800 μg (triangles) or vehicle (circles) was injected intratumorally on day 8 to B16-SIY flank tumors and tumor size was measured by caliper. Data are mean ± s.e.m. (*n* = 9 mice in each group). **d**, NVS-STG2 induces significant IFNγ responses at 6 h after dosing by one-way analysis of variance (ANOVA), with *P* = 0.0008 (400 μg) and *P* < 0.0001 (800 μg) in Dunnett’s multiple comparisons test (mean ± s.d., *n* = 9 mice in each group). **e**, NVS-STG2 (800 μg) induces significant anti-SIY tumor T cell response at 6 days after treatment by one-way ANOVA, with *P* = 0.0009 in Dunnett’s multiple comparisons test (lines represent medians in scatter plots, *n* = 8 mice in each group). ***P < 0.001; ****P < 0.0001; NS, not significant (*P* > 0.05). Source data

To determine the potential antitumor efficacy, MC38 tumors were inoculated into the hSTING knock-in mice. NVS-STG2 was injected intratumorally on days 11, 14 and 18. NVS-STG2 slowed tumor growth significantly, and four of nine mice saw no tumor growth during the 33-day experimental period (Fig. 4a,b).

Host IFN signaling is important for the development of an antitumor CD8^+^ T cell response and for the antitumoral efficacy of CDN STING agonists^42,45,46^. We also evaluated the tumor-specific T cell response stimulated by NVS-STG2 in hSTING knock-in mice bearing B16-SIY tumors^47^ (Fig. 4c). After tumor implantation, the tumors grew for 8 days before a single treatment with NVS-STG2. At 6 h after dosing, a significant increase in plasma IFNγ level was observed with NVS-STG2 treatment (Fig. 4d). T cell responses against the SIY peptide were measured 5 days after the treatment. NVS-STG2 exhibited a dose-dependent and significant induction of T cell priming response (Fig. 4e).

## Discussion

STING has been of high interest for pharmacological intervention. By using a combination of functional screens, biochemical dissection and structural characterization, we identified NVS-STG2 as a STING agonist that binds at the TMD interface of STING, thereby acting as a molecular glue to promote STING oligomerization. It is tempting to speculate that there could be a cellularly endogenous ligand(s) that binds to the NVS-STG2-binding site and contributes to STING activation, independently or together with cGAMP. Cholesterol and lipids are known to modulate STING signaling and it is conceivable that such metabolites may engage the binding site of NVS-STG2 (refs. ^48–50^). Our multiple cryo-EM structures of hSTING bound to various combinations of cGAMP, NVS-STG2 and C53 show nearly identical structures of the high-order oligomerization, suggesting potential crosstalk and cooperativity across the ligand-binding sites in regulating STING activation. The identification of another agonist-binding site provides potential opportunities for STING activation to enable the development of better therapeutic STING agonists. The structures provide a basis for generating more potent agonists of STING targeting the NVS-STG2-binding pocket by modifying NVS-STG2 or starting with new scaffolds that fit this pocket. Bulky compounds that can bind this pocket but disallow the side-by-side packing required for the high-order oligomerization may act as antagonists of STING, and may be used as therapeutics for STING-related autoimmune diseases.

## Methods

### Cells and cell culture

THP1-Dual cells (Invivogen, catalog (cat.) no. thpd-nfis) and THP1 cells were cultured in RPMI complete medium (RPMI1640 containing l-glutamine; Invitrogen, cat. no. 11875-085), 10% v/v FBS (Invitrogen, cat. no. 26400-044) and penicillin-streptomycin (Pen/Strep, Invitrogen, cat. no. 15070-063) at 37 °C, +5% CO_2_. The MC38 cell line derived from C57BL/6 murine colon adenocarcinoma was acquired from the laboratories of James Hodge, PhD and Jeffery Schlom, PhD (National Cancer Institute, National Institutes of Health). The murine melanoma-derived B16-SIY cells (referred to as B16-SIY; engineered to express the model SIYRYYGL (SIY) antigen to enable immune monitoring) were acquired from the Gajewski lab (University of Chicago)^47^. FreeStyle 293-F cells were acquired from Thermo Fisher (cat. no. R79007). Cell lines were regularly tested and maintained negative for mycoplasma.

### Primary high-throughput screen

THP1-Dual cells in RPMI complete growth medium were dispensed in a Washer/Dispenser II (GNF Systems) at 7,500 cells per 5 µl in a Greiner 1536-well white-bottom plate (Greiner, cat. no. 789173A) and incubated overnight at 37 °C, +5% CO_2_. In total, ~250,000 compounds from the Novartis screening deck was tested at a final concentration of 50 µM using an Echo555 dispenser (Labcyte). The plates were incubated for 24 h at 37 °C and 5% CO_2_. The plates were set at room temperature for 15 min before a Washer/Dispenser II (GNF Systems) was used to add 5 µl of a QUANTI-Luc (Invivogen, cat. no. rep-qlc-20) solution and the plates were immediately read on a ViewLux (PerkinElmer) using a 5-s luminescence read. The active control on the plate, 50 µM of 2′,3′-cGAMP, was used to define the +100% maximum reporter activity. The neutral control, 0.45% DMSO, was defined as 0%. The average overall luminescence signal (*µ*) value was 0.18 with 2.94 standard deviation (*σ*). With a cutoff of activity relative to the 2′,3′-cGAMP active control, the overall hit rate was 0.25%.

### Reporter assay with STING co-transfection in HEK293T cells

All experiments were independent biological replicates. Equal volumes (6.6 µl) of 3.24 µg µl^−1^ p5xISRE-IFNβ-GL4 and 0.05 µg µl^−1^ complementary DNA STING construct were mixed and diluted into 500 µl of a FuGENE 6 (Promega, cat. no. E2691) solution diluted (1:30) into Opti-MEM (Thermo Fischer, cat. no. 31985070). Trypsinized HEK293T cells were diluted to 0.35 × 10^6^ cells per ml into DMEM complete growth medium and 16.25 ml of the cells was added to the DNA/FuGENE 6 mixture. Then, 25 µl of the transfected cells were transferred to a Black Grenier 384-well µCLEAR plate (Thermo Fisher, cat. no. 781091) and incubated for 24 h (37 °C, +5% CO_2_). Compounds were then Echo dispensed (100 nl; Beckman Coulter, Echo 525) into the appropriate wells and incubated for 24 h (37 °C, +5% CO_2_). Bright-Glo (25 µl, Promega, cat. no. E2620) was added and the plate was incubated at room temperature for 5 min. Luminescence was then read on a ViewLux (PerkinElmer) using program 6. The active control on the plate, 50 µM of 2′,3′-cGAMP, was used to define the +100% reporter activity. The neutral control, 0.45% DMSO, was defined as 0%.

### STING translocation/aggregate assay

The pCMV6-hSTING-GFP plasmid was from Origene (cat. no. RG208418). The hSTING R95A mutation was generated using Q5 Site-Directed Mutagenesis Kit (New England BioLabs, cat. no. E0554S). Mutation forward primer: CTACTCCCTCCCAAATGCGGTC; and reverse primer: GCGAAATAGATGGACAGCAGCAACAG. Both hSTING and hSTING(R95A) plasmids were linearized using restriction endonuclease Sca I (Thermo Fisher, cat. no. ER0431) and gel purification. Linearized plasmid DNA was transfected into 293T cells using FuGene 6 Transfection Reagent (Promega, cat. no. E2691). 293T_hSTING and hSTING(R95A) GFP-positive cells were sorted using Flow cytometry.

First, 100,000 293T stable cells were plated in each well of a chambered coverslip with eight individual wells (ibidi, cat. no. 80809). Cells were treated with either DMSO and 50 μM 2′,3′-cGAMP or 50 μM NVS-STG2 overnight. Images were taken under a ZEISS microscope with a 20× objective.

### Western blots

Cells were collected and washed with PBS, then lysed with RIPA buffer (Thermo Fisher, cat. no. 89900) plus Halt Protease and Phosphatase Inhibitor Cocktail (Thermo Fisher, cat. no. 1861281) for 20 min on a shaker at 4 °C. Cell lysates were cleared by centrifuging at ≥14,000 r.p.m. (20,000*g*) for 10 min at 4 °C. Protein concentration was determined using a protein assay kit (Bio-Rad, DC Protein Assay Reagents). Equal amounts of proteins from cleared cell lysates were mixed with NuPAGE LDS Sample Buffer (Invitrogen, cat. no. NP0007) and NuPAGE Sample Reducing Agent (Invitrogen, cat. no. NP0009), heated for 10 min at 95 °C, then loaded and electrophoresed on a 4–20% gradient Tris-glycine gel (Bio-Rad Criterion TGX Precast Gel). The protein was electrophoretically transferred to a 0.2-µm nitrocellulose membrane (Bio-Rad) by Bio-Rad Trans-Blot Turbo Transfer System. The membrane was then blocked with blocking reagent (5% BSA in TBS-Tween-20 0.05%) for 1 h. After blocking, the membrane was incubated with pIRF3 antibody (Ser396) (4D4G) (Cell Signaling Technology, cat. no. 4947) diluted 1:1,000 in blocking reagent overnight at 4 °C. The membrane was washed with TBS-Tween-20 0.05%, then incubated with HRP-Goat anti-Rabbit IgG (H + L) secondary antibody (Bio-Rad, cat. no. 1706515) for 1 h at room temperature. The membrane was washed with TBS-Tween-20 0.05%, then the membrane was soaked in chemiluminescent substrate (Thermo Fisher, cat. no. 34076) for 5 min and exposed with GE ImageQuant TL LAS 4000 or X-Ray film to visualize the pIRF3 band.

### Expression of hSTING-LBD (V155-V341)

Sequences for hSTING proteins were codon-optimized for *Escherichia* *coli* expression and included a C-terminal 6 × HIS tag. The sequences were synthesized (GeneArt) and directly ligated into linearized pDEST14 (Invitrogen) resulting in an expression plasmid lacking expressed Gateway linker sequences. The plasmid was transformed into BL21(DE3) cells (Invitrogen) and a 1 l culture grown in terrific broth (TB) medium was induced with 1 mM IPTG for 3 h at 37 °C. The induced culture was pelleted, resuspended in lysis buffer (Qiagen Qproteome, cat. no. 37900) and clarified by centrifugation at 16,000*g* for 20 min. The clarified lysate was purified over a 5 ml HisTrap FF column (Cytiva, cat. no. 17531901) using standard buffers. The peak elution fractions were pooled and further purified by size-exclusion chromatography using a HiLoad 16/660 Superdex 75 pg (Cytiva, cat. no. 28-9893-33) in buffer containing 150 mM NaCl, 50 mM Tris pH 8 and 10% glycerol. STING migrated as a single peak consistent with a dimer of apparent molecular weight of about 45 kDa. Peak fractions were combined and tested for activity by DSF.

### DSF assay

All experiments were independent experiments of technical replicates. First, 10 µl of 20 µM hSTING (V155-V341) protein in 1 M HEPES pH 7.5 per 5 M NaCl was mixed with 200 µM of compound and 10 µl of 10 × Sypro orange (Thermo Fischer) in 384-well PCR plates (Roche, cat. no. 04729749001). Plates were sealed and centrifuged at 2,000 r.p.m. (800*g*) for 2 min. A protocol using a 25–80 gradient at 0.5 °C ramp in a Bio-Rad C1000/CFX384 thermocycler was used to obtain the raw fluorescence data which were analyzed using the extended Boltzman model to define the melting temperature.

### PAL and competition experiment

In brief, 1 × 10^7^ THP1 cells were added to individual 15-cm^2^ plates and allowed to recover overnight at 37 °C with 5% CO_2_. The following day, each plate was washed three times with PBS and incubated in Opti-MEM medium. DMSO was added to five plates, while the remaining two plates were treated with 20 µM NVS-STG3 for 1 h at 37 °C with 5% CO_2_. DMSO- and NVS-STG3-treated cells were then treated with 1 µM of compound NVS-STG4 for 1 h at 37 °C with 5% CO_2_. Irradiation of the plated cells followed by cell lysis, enrichment, sample processing and data acquisition were performed as previously described^29^.

### RT–qPCR analysis of cytokines

All experiments were done as independent experiments of biological replicates. Total RNA was isolated using the RNeasy kit (QIAGEN) and incubated with DNase I, Amplification Grade (Invitrogen). cDNA was synthesized using High-Capacity cDNA Reverse Transcription Kit (Applied Biosystems), and expression of cytokines was measured by real-time RT–qPCR using specific primers/probes for mouse *Ifnb*. PCR reactions were performed in the 7300 Real Time PCR system (Applied Biosystems). The results are expressed as 2^−Δ*C*t^ using *18S* as an endogenous control (*Ifnb* forward primer: GGAAAGATTGACGTGGGAGA; reverse primer: CCTTTGCACCCTCCAGTAAT; probe: CTGCTCTC).

### In vivo methods

#### General

All animal experiment procedures were performed according to the guidelines approved by the Institutional Animal Care and Use Committee (IACUC) of Novartis, Cambridge, MA and following the guidance of the Association for Assessment and Accreditation of Laboratory Animal Care (AAALAC). Tumor and bodyweight measurements were performed using calipers and a weigh scale, respectively. Mice were euthanized when tumor volume approached ~2,000 mm^3^, weight loss exceeded 20% or tumors ulcerated. When necessary, plasma and tumor samples were collected at specific time points and frozen for analysis.

#### Animals

Female C57BL/6 wild-type and hSTING knock-in mice (10–12 weeks old, 20–25 g) were purchased from the Jackson Laboratory or bred in-house. All animal protocols were approved by the IACUC of Novartis, Cambridge, MA, following the guidance of the AAALAC.

#### CRISPR generation of hSTING knock-in mice

A single guide RNA (sgRNA) targeting the start ATG in exon 3 of *Tmem173* was identified with a previously available online design tool from the Zhang lab. This sgRNA sequence (5′-CAGTAGTCCAAGTTCGTGCG) was cloned into pSpCas9(BB)^51^. The sgRNA for zygote injection was generated using the MEGAshortscript T7 transcription kit (Invitrogen, cat. no. AM1354), and was then purified with the MEGAclear transcription clean-up kit (Invitrogen, cat. no. AM1908). The targeting vector was generated by including 3 kb of mouse genomic sequence upstream of the ATG as the 5′ homology arm, and 2 kb of mouse genomic sequence (starting 24 bp before the end of exon 3) as the 3′ homology arm. The wild-type human *TMEM173* cDNA sequence, followed by a polyA sequence and an FRT-flanked Neo selection cassette, was cloned in between the 5′ and 3′ homology arms. Before microinjection, the targeting vector was linearized and purified. Linearized plasmid, purified sgRNA and Cas9 protein (PNA Bio) were pre-mixed on ice (125 ng μl^−1^ sgRNA, 125 ng μl^−1^ Cas9 protein and 2.5 ng μl^−1^ linearized donor) before microinjection into fertilized C57BL/6J oocytes.

#### Genotyping

Knock-in founder mice were identified by nested PCR screening around both homology arm regions. For the 5′ arm, the first PCR was with primers 628 (5′-CTCACTGGGTGGAGCACTAA) and 629 (5′-CGGTACCTGGAGTGGATGTG), with subsequent amplification using primers 632 (5′-CCAGCTGAGGCAGGGTTTAT) and 633 (5′-GTACCGGAGAGTGTGCTCTG). For the 3′ arm, the primers used for the first PCR were 902 (5′-ATCGTCTGTTGTGCCCAGTCATAG) and 907 (5′-GCTTGGGTTACATATTGAGACCCTG). The next round of amplification was performed with primers 903 (5′-GCAATCCATCTTGTTCAATGGCCG) and 906 (5′-TCAGCGGTAAAGAGCACCTGCTG). The resulting PCR products were Sanger sequenced to confirm targeting to the appropriate location. MEF lines derived from two knock-in founders were subsequently analyzed by Southern blot using three probes, with the data indicating that one founder line was correctly targeted but with multiple copies of the targeting vector integrated in tandem. This multi-copy integration event was found to result in high levels of expression of hSTING. Breeding the line with a mouse strain expressing FLPe in the germline removed all but one copy of the human cDNA, and resulted in a strain with lower hSTING expression.

#### BMDM cells

BMDM cells from wild-type, *STING1*^−/−^ and hSTING knock-in mice were generated by culturing cells from the tibias and femurs in DMEM containing 10% FBS and 1% penicillin/streptomycin in the presence of rmGM-CSF (20 ng ml^−1^; BioLegend) for 9 days at 37 °C and 5% CO_2_.

#### Animal flank tumor models

Tumor cells were inoculated subcutaneously into the lower flank with 0.5 × 10^6^ tumor cells in 100 µl of serum-free DMEM or RPMI1640. The flank tumors reached an average size of 70–100 mm^3^ (10–12 days) when vehicle or NVS-STG2 was injected intratumorally, totaling three times (3–4 days apart). At 6 h after dosing, mice were bled for cytokine analysis and tumor growth was calipered and monitored. At 6 days after treatment, PBMC ELISPOT was performed.

#### Mouse PBMC isolation

PBMCs were isolated using Lymphocyte-Mammal Cell Separation Media from mouse whole blood.

#### IFNγ ELISPOT

All experiments were independent biological replicates. ELISPOT was conducted with the R&D Systems mouse IFNγ kit (cat. no. EL485) according to the provided protocol. In brief, ELISPOT plates were coated with mouse IFNγ antibody. Splenocytes or PBMCs from tumor-challenged mice were isolated and plated at 2 × 10^5^ per well. PBMCs were stimulated with 5 μM SIY peptide (SIYRYYGL), then plates were incubated at 37 °C for 48 h. Next, plates were washed, and detection antibody was added and incubated for 2 h at room temperature. Streptavidin-AP concentrate was added for 2 h at room temperature, followed by addition of BCIP/NBT substrate. Developed plates were dried overnight, read using an ImmunoSpot S6 Micro Analyzer and analyzed with ImmunoSpot software.

#### Mouse serum cytokines

Mice were bled 6 h after intratumoral injection of NVS-STG2. IFNγ was measured by the MSD V-plex proinflammatory panel 1 kit (MSD Multi-Spot Assay System).

### Expression and purification of full-length hSTING

As described previously, the coding sequence of hSTING (residues 1–343) followed by an HRV-3C protease recognition sequence and the purification tag Tsi3 protein coding sequence was inserted into pEZT-BM vector^33^. The protein was expressed in Freestyle 293-F cells by the BacMam system^52^. In brief, the hSTING construct was transfected to the *E. coli* strain DH10Bac to produce the bacmid. The bacmid was transfected to sf9 cells to produce and amplify baculovirus. Freestyle 293-F cells were infected with the baculovirus at 3:100 (v:v) ratio, and cultured at 37 °C for 50 h for protein expression. Sodium butyrate at 4 mM was used to boost expression. All of the purification steps were performed at 4 °C. Cells were spun down and resuspended in buffer A containing 20 mM Tris-HCl, pH 8.0, 200 mM NaCl, 1 mM DTT and protease inhibitor cocktail (Roche). Cells were disrupted by a high-pressure cell disruptor and the membrane fraction was obtained by ultracentrifugation at 150,000*g* for 1 h. The pellet was resuspended in buffer A supplemented with 1% (w/v) *n*-dodecyl-β-d-maltopyranoside (DDM) and 0.2% cholesteryl hemisuccinate Tris salt (CHS) (Anatrace) to extract membrane proteins. After ultracentrifugation at 100,000*g* for 1 h, the supernatant was supplemented with 1 mM CaCl_2_ and loaded to Tse3-conjugated Sepharose resin^12^. The resin was washed with 15 column volumes of buffer A supplemented with 0.06% DDM, 0.006% CHS and 1 mM CaCl_2_. hSTING was eluted by HRV-3C cleavage. The protein was further purified by the size-exclusion column Superdex 200 Increase 10/300GL (Cytiva) in buffer B (20 mM HEPES-Na, pH 7.5, 150 mM NaCl, 0.03% DDM and 0.003% CHS). The main peak was pooled (~12.5 µM) and incubated with cGAMP and NVS-STG2 at 1:2:2 molar ratio or cGAMP, c53 and NVS-STG2 at 1:2:2:2 molar ratio overnight. The samples were then concentrated to 4–6 mg ml^−1^ for cryo-EM grid preparation.

### Cryo-EM data collection and image processing

The samples of STING in complex with either cGAMP/NVS-STG2 or cGAMP/C53/NVS-STG2 were applied to glow-discharged Quantifoil R1.2/1.3 300-mesh gold holey carbon grids (Quantifoil, Micro Tools), blotted under 100% humidity at 4 °C and frozen by plunging into liquid ethane using a Mark IV Vitrobot (FEI). Micrographs were collected in the super-resolution counting mode on a Titan Krios microscope (FEI) with a K3 Summit direct electron detector (Gatan), with the slit of the GIF-Quantum energy filter set to 20 eV. The nominal magnification was ×81,000, corresponding to the pixel size of 1.08 Å. Micrographs were dose-fractioned into 36 frames. The total exposure time was 7.2 s and the dose rate was ~1.6 e^−^ Å^−2^ per frame in the correlated double sampling mode. Motion-correction and dose-weighting were applied using the Motioncor2 program (v.1.2)^53^. GCTF 1.06 was used for contrast transfer function (CTF) correction^54^. Particles were picked using templated-based picking in RELION v.4.0 (ref. ^55^). All of the following image processing steps were carried out in RELION as well. For the dataset of STING bound to cGAMP/NVS-STG2, particles were extracted with the box size of 160 pixels, which was large enough to accommodate four STING dimers, and binned by a factor of 4 for two-dimensional (2D) classification. The box size was 216 pixels for the dataset of STING bound to cGAMP/NVS-STG2. The 2D and 3D classification steps were done as outlined in Extended Data Figs. 2 and 3 to select good particles for 3D refinement with the *C*2 symmetry. The cryo-EM map of STING bound to cGAMP/C53 (EMD ID EMD-25142) was low-pass filtered and used as the initial mode. Bayesian polishing and CTF refinement were used at the final stage to improve the resolution of the maps. Resolution was estimated with the Fourier shell correlation 0.143 criterion. Local resolution of the maps was estimated in RELION.

### Model building and refinement

Model building of both the cGAMP/C53/NVS-STG2-bound and the cGAMP/NVS-STG2-bound structures was initiated by docking the previous structure of hSTING tetramer bound to cGAMP and C53 (PDB ID 7SII) into the cryo-EM maps, followed by manual building in Coot v.0.98 (ref. ^56^). Both the structures were of sufficient quality for modeling most of the residues in hSTING and confirmed that the dimer adopts the 180-degree rotated conformation of the LBD relative to the TMD as seen in the previous active structures of STING^33,57^. The high-resolution map (2.95 Å) of the cGAMP/C53/NVS-STG2-bound structure allowed two NVS-STG2 molecules to be placed unambiguously into the TMD–TMD cavity between the two STING dimers, adjacent to the two-fold symmetry axis of the tetramer. Based on this assignment, NVS-STG2 was modeled in the lower-resolution structure of STING bound to cGAMP/NVS-STG2. C53 and cGAMP from the previous cGAMP/C53-bound structure fit well to the new cryo-EM densities without much adjustment. The only difference was that the previous structure was calculated from particles containing mostly STING tetramers, where the two dimers bound to C53 in a symmetric manner^33,57^. The STING samples with either cGAMP/C53/NVS-STG2 or cGAMP/NVS-STG2 bound formed much longer high-order oligomers as shown in Fig. 2b. Tetrameric particles extracted from these samples were mostly segments from longer oligomers. C53 therefore could in principle adopt the two alternative binding orientations in the TMD pocket of the STING dimer with roughly equal probability, leading to an O-shaped density, rather than the C-shape seen in the previous structure (Extended Data Fig. 4). We chose one orientation of C53 arbitrarily to fit the density. Real-space refinement of the model was carried out with Phenix v.1.18 (ref. ^58^). Molprobity as a part of the Phenix validation tool was used for assessing the quality of the model^58^. Statistics of the refined model are summarized in Supplementary Table 1. Structural figures were rendered in Chimera v.1.16 (ref. ^59^).

### Native gel analyses of STING oligomerization

Purified hSTING protein at 20 µM in buffer B was incubated with the compounds (cGAMP, c53, NVS-STG2 or their different combinations) at 40 µM at 4 °C overnight. The samples were mixed with the native gel sample buffer (Invitrogen, cat. no. BN20032) and resolved by a 3–12% gradient native gel (Invitrogen, cat. no. BN2012BX10).

### Graph generation and statistical analysis

GraphPad Prism v.9.5 was used for generating graphs and statistical analysis.

### Reporting summary

Further information on research design is available in the Nature Portfolio Reporting Summary linked to this article.

## Online content

Any methods, additional references, Nature Portfolio reporting summaries, source data, extended data, supplementary information, acknowledgements, peer review information; details of author contributions and competing interests; and statements of data and code availability are available at 10.1038/s41589-023-01434-y.

### Supplementary information

Supplementary Information Chemical synthesis procedure and Supplementary Table 1.

Reporting Summary

Supplementary Data 1 STING agonist chemoproteomics raw data.

### Source data

Source Data Fig. 1 Statistical source.

Source Data Fig. 1 Uncropped and unprocessed gels.

Source Data Fig. 2 Uncropped and unprocessed gels.

Source Data Fig. 3 Statistical source.

Source Data Fig. 4 Statistical source.

Source Data Extended Data Fig. 1 Statistical source.

Source Data Extended Data Fig. 6 Uncropped and unprocessed gels and raw images.

Source Data Extended Data Fig. 7 Statistical source.

## Data Availability

The atomic coordinates and the cryo-EM map of STING bound to cGAMP/NVS-STG2 have been deposited to the RCSB Protein Data Bank (PDB) (accession 8FLK) and the Electron Microscopy Data Bank (EMDB) (accession EMD-29281), respectively. The coordinates and map of STING bound to cGAMP/NVS-STG2/C53 have been deposited to the PDB and EMDB with accession numbers 8FLM and EMD-29282, respectively. Source data are provided with this paper.
