# Supplementary material for: Activation of human STING by a molecular glue-like compound
Source: Nat Chem Biol. 2023 Oct 12;20(3):365–72. doi: 10.1038/s41589-023-01434-y (PMC10907298; doi:10.1038/s41589-023-01434-y)
Supplement: Supplementary file 1 — Chemical synthesis procedure and Supplementary Table 1. [file 41589_2023_1434_MOESM1_ESM.pdf]

nature chemical biology

Article

https://doi.org/10.1038/s41589-023-01434-y

Activation of human STING by a molecular glue-like compound

In the format provided by the authors and unedited

Supplementary information:

Chemical synthesis procedure

Supplementary Table 1

Chemical Synthesis

General information: Unless otherwise noted, all reagents were purchased from commercial sources with purity ≥95% and used without further purification. NMR spectra were recorded on a Bruker Avance II 400 MHz spectrometer. All chemical shifts are reported in parts per million (δ) relative to tetramethylsilane. The following abbreviations are used to denote signal patterns: s = singlet, d = doublet, t = triplet, m = multiplet, p = pentet, and br = broad. Low resolution mass spectra were recorded using an Agilent 1100 series LC-MS spectrometer. High resolution mass spectra were recorded using a Waters AcQuity UPLC coupled to a Waters Xevo G2-XS Tof mass spectrometer. Flash chromatography was conducted using grade 60 230−400 mesh silica gel from Fisher Chemical (S825-1) or by utilizing the CombiFlash Companion from Teledyne Isco, Inc. and RediSep Rf disposable normal phase silica gel columns (4−300 g). Thin layer chromatography was performed using 2.5 × 7.5 cm glass-backed TLC Silica Gel 60 F254 plates from EMD Chemicals, Inc. (15341-1) and visualized by UV light. The purity of all synthesized compounds was ≥95%, unless otherwise noted.

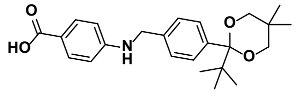

NVS-STG1 (1)

NVS-STG1 was previously synthesized in an analogous manner to NVS-STG2, as below.

^{1}H NMR (400 MHz, DMSO) δ 11.99 (s, 1H), 7.69 – 7.62 (m, 2H), 7.38 (d, *J* = 7.9 Hz, 2H), 7.22 (d, *J* = 8.0 Hz, 2H), 7.00 (t, *J* = 5.9 Hz, 1H), 6.68 – 6.59 (m, 2H), 4.37 (d, *J* = 5.9 Hz, 2H), 3.35 (d, *J* = 11.0 Hz, 2H), 3.22 (d, *J* = 10.8 Hz, 2H), 1.17 (s, 3H), 0.86 (s, 9H), 0.47 (s, 3H).

^{13}C NMR (101 MHz, DMSO) δ 167.44 (C), 152.54 (C), 138.78 (C), 133.89 (C), 131.10 (CH), 129.49 (CH), 126.48 (CH), 117.16 (C), 111.04 (CH), 103.75 (C), 70.74 (CH_2), 45.68 (CH_2), 29.56 (C), 24.79 (CH_3), 22.64 (CH_3), 21.66 (CH_3).

HRMS-ESI (*m/z*) $[M+H]^+$ calculated for $C_{24}H_{32}NO_4$, 398.2331; found, 398.2335.

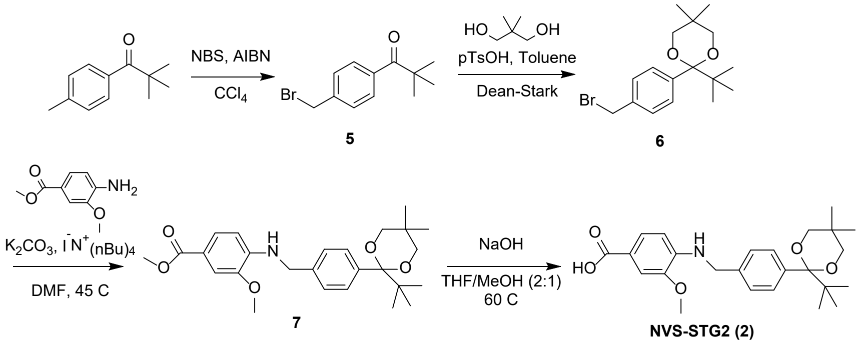

1-(4-(Bromomethyl)phenyl)-2,2-dimethylpropan-1-one (5)

2,2-Dimethyl-1-(4-methylphenyl)propane-1-one (CAS: 30314-44-4; 29.0 g, 165 mmol), N-bromosuccinimide (29.3 g, 165 mmol) and AIBN (2.70 g, 16.5 mmol) were combined in carbon tetrachloride (235 mL) and heated to 100 ºC for 5 h. The reaction mixture was then cooled to room temperature and concentrated under reduced pressure. The slurry was purified by filtering through a plug of silica gel and eluting with 10% EtOAc in heptanes to elute a yellow oil (43.0 g, 100%). ^{1}H NMR (400 MHz, $CDCl_3$) δ 7.70 – 7.66 (m, 2H), 7.47 – 7.37 (m, 2H), 4.50 (s, 2H), 1.35 (s, 9H).

2-(4-(Bromomethyl)phenyl)-2-(*tert*-butyl)-5,5-dimethyl-1,3-dioxane(6)

1-(4-(Bromomethyl)phenyl)-2,2-dimethylpropan-1-one (43.0 g, 169 mmol), 2,2-dimethylpropane-1,3-diol (26.3 g, 253 mmol), and pTsOH (1.60 g, 8.43 mmol) were combined and suspended in toluene (250 mL). The flask was fitted with a Dean–Stark trap and was heated to 130 ºC for 16 h. The reaction mixture was cooled to room temperature and Celite® was added and concentrated under reduced pressure. The solid was purified by silica gel column chromatography (0-15% EtOAc/heptanes) to afford the product as a white solid (46.0 g, 99%). ^{1}H NMR (400 MHz, $CDCl_3$) δ 7.41 – 7.37 (m, 2H), 7.33 – 7.29 (m, 2H), 4.54 (s, 2H), 3.34 (s, 4H), 1.24 (d, *J* = 4.6 Hz, 4H), 0.92 (s, 9H), 0.54 (s, 3H).

Methyl 4-((4-(2-(*tert*-butyl)-5,5-dimethyl-1,3-dioxan-2-yl)benzyl)amino)-3-methoxybenzoate(7)

2-(4-(Bromomethyl)phenyl)-2-(tert-butyl)-5,5-dimethyl-1,3-dioxane (12.0 g, 35.2 mmol), 4-amino-3-methoxybenzonitrile (6.69 g, 36.9 mmol), and potassium carbonate (19.4 g, 141 mmol) were suspended in DMF (88 mL). Tetrabutylammonium iodide (13.0 g, 35.2 mmol) was added at room temperature and stirred for 1 h, the reaction was then heated to 45 ºC and maintained for 16 h. The starting material was consumed, and the reaction mixture was cooled and partitioned between 1N HCl and EtOAc. The aqueous layer was extracted with EtOAc (2 × 50 mL) and the combined organic layers were washed with brine and concentrated to an amber oil. The oil residue was purified by silica gel column chromatography and eluted with 0-40% EtOAc/heptanes to isolate a white solid (6.83 g, 44%). ^{1}H NMR (400 MHz, DMSO) δ 7.41 (dt, *J* = 8.4, 1.9 Hz, 1H), 7.36 – 7.32 (m, 2H), 7.30 (t, *J* = 1.9 Hz, 1H), 7.22 – 7.16 (m, 2H), 6.51 (dd, *J* = 8.4, 1.5 Hz, 1H), 6.44 (d, *J* = 6.1 Hz, 1H), 4.44 (d, *J* = 5.9 Hz, 2H), 3.87 (d, *J* = 1.7 Hz, 3H), 3.74 (d, *J* = 1.4 Hz, 3H), 3.34 (d, *J* = 11.3 Hz, 2H), 3.21 (d, *J* = 10.7 Hz, 2H), 1.16 (s, 3H), 0.84 (d, *J* = 2.0 Hz, 10H), 0.46 (s, 3H).

NVS-STG2 (2)

Methyl 4-((4-(2-(tert-butyl)-5,5-dimethyl-1,3-dioxan-2-yl)benzyl)amino)-3-methoxybenzoate (9.06 g, 20.52 mmol) was dissolved in THF (137 mL) and MeOH (68.0 mL) was added as a co-solvent. A 1N NaOH solution (2.46 g, 61.6 mmol, 61.5 mL) was added to the mixture which was then refluxed for 12 h. The reaction was cooled to room temperature and concentrated under reduced pressure. The mixture was resuspended in water (50 mL) and cooled to 0 ºC. The mixture was acidified with a 10% solution of citric acid (~17 mL) to obtain a pH of ~7. The solid was filtered, washed with water followed by washing with ether and finally pentanes to afford an off-white solid (8.22 g, 94%)

^{1}H NMR (400 MHz, Chloroform-d) δ 7.63 (dd, J = 8.4, 1.4 Hz, 1H), 7.40 (d, J = 1.5 Hz, 1H), 7.30 - 7.21 (m, 4H), 6.54 (d, J = 8.6 Hz, 1H), 5.13 (s, 1H), 4.38 (s, 2H), 3.86 (s, 3H), 3.27 (s, 5H), 1.17 (d, J = 5.6 Hz, 5H), 0.85 (s, 10H), 0.46 (s, 3H).

^{1}H NMR (400 MHz, DMSO) δ 7.39 – 7.33 (m, 3H), 7.28 (dd, *J* = 8.2, 1.6 Hz, 1H), 7.23 – 7.12 (m, 2H), 6.37 (d, *J* = 8.0 Hz, 1H), 5.53 (t, *J* = 6.2 Hz, 1H), 4.35 (d, *J* = 5.9 Hz, 2H), 3.80 (s, 3H), 3.33 (s, 6H), 3.23 (d, *J* = 10.9 Hz, 3H), 1.16 (s, 3H), 0.85 (s, 9H), 0.47 (s, 3H).

^{13}C NMR (101 MHz, DMSO) δ 169.85 (C), 144.98 (C), 139.78 (C), 138.63 (C), 133.59 (C), 129.40 (CH), 126.27 (CH), 122.53 (CH), 110.74 (CH), 107.72 (CH), 103.77 (C), 70.75 (CH_2), 55.14 (CH_3), 46.06 (CH_2), 29.57 (C), 24.82 (CH_3), 22.68 (CH_3), 21.67 (CH_3).

HRMS-ESI (*m/z*) $[M+H]^+$ calculated for $C_{25}H_{34}NO_5$, 428.2436; found, 428.2488.

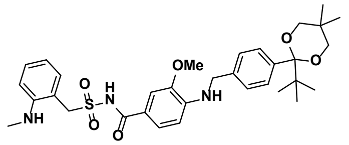

NVS-STG3 (3)

NVP-STG2 (0.100 g, 0.234 mmol), EDCI (0.068 g, 0.351 mmol), (2-(methylamino)phenyl)methanesulfonamide (0.071 g, 0.351 mmol) and DMAP (0.043 g, 0.351 mmol) were combined in DCE (2 mL) and heated to 40 °C for 5h. The reaction was cooled to room temperature and was concentrated under reduced pressure. The crude reaction mixture was filtered and purified directly by preparative reverse-phase HPLC (Column: Waters X-Bridge, C18 OBD, 5 μm, 30 × 50 mm, 75 mL/min, 55-80% acetonitrile:water (both containing 0.1% formic acid) gradient over 14 min). The isolated fractions were lyophilized to afford a white solid (0.032 g, 22%).

^{1}H NMR (400 MHz, $CDCl_3$) δ 7.42 – 7.36 (m, 1H), 7.35 – 7.29 (m, 3H), 7.24 (d, *J* = 4.0 Hz, 5H), 6.48 (d, *J* = 8.3 Hz, 1H), 4.94 (s, 2H), 4.34 (s, 2H), 3.79 (s, 3H), 3.26 (s, 4H), 3.05 (s, 3H), 2.68 (s, 1H), 1.15 (s, 3H), 0.84 (s, 9H), 0.44 (s, 3H).

^{13}C NMR (101 MHz, $CDCl_3$) δ 166.71 (C), 146.28 (C), 143.69 (C), 139.94 (C), 137.08 (C), 135.87 (C), 133.88 (CH), 131.56 (CH), 130.58 (CH), 127.60 (CH), 126.60 (CH), 123.87 (CH), 120.91 (CH), 120.04 (C), 117.16 (C), 108.88 (CH), 108.33 (CH), 104.65 (C), 71.65 (CH_2), 56.22 (CH_2), 55.85 (CH_3), 47.12 (CH_2), 39.89 (CH_3), 35.93 (CH_3), 30.13 (C), 25.08 (CH_3), 23.19 (CH_3), 22.18 (CH_3).

HRMS-ESI (*m/z*) $[M+H]^+$ calculated for $C_{33}H_{44}N_3O_6S$, 610.2951; found, 610.2999.

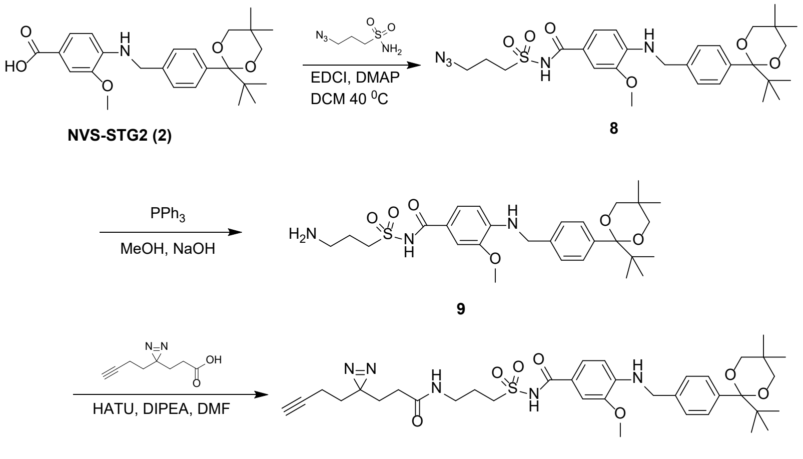

NVS-STG2 (2) **8** **9** **NVS-STG4 (4)**

N-((3-azidopropyl)sulfonyl)-4-((4-(2-(tert-butyl)-5,5-dimethyl-1,3-dioxan-2-yl)benzyl)amino)-3-methoxybenzamide (8)

NVP-STG2 (0.200 g, 0.468 mmol), EDCI (0.135 g, 0.702 mmol), 3-azidopropane-1-sulfonamide (0.084 g, 0.515 mmol) and DMAP (0.086 g, 0.702 mmol) were combined in DCM (1 mL) and heated to 40 °C for 16h. The reaction was cooled to room temperature and was concentrated under reduced pressure. The residue was purified by silica gel column chromatography and eluted with 0-10% MeOH/DCM to isolate an off-white foam (0.226 g, 84%).

^{1}H NMR (400 MHz, $CDCl_3$) δ 7.41 (dd, *J* = 8.3, 2.0 Hz, 1H), 7.36 (d, *J* = 1.9 Hz, 1H), 7.31 (s, 4H), 6.58 (d, *J* = 8.4 Hz, 1H), 5.23 (t, *J* = 5.6 Hz, 1H), 4.43 (d, *J* = 5.2 Hz, 2H), 3.92 (s, 3H), 3.69 – 3.60 (m, 2H), 3.48 (d, *J* = 6.6 Hz, 4H), 3.26 – 3.18 (m, 2H), 2.20 – 1.98 (m, 2H), 1.23 (s, 3H), 0.92 (s, 9H), 0.52 (s, 3H).

N-((3-aminopropyl)sulfonyl)-4-((4-(2-(tert-butyl)-5,5-dimethyl-1,3-dioxan-2-yl)benzyl)amino)-3-methoxybenzamide (9)

N-((3-azidopropyl)sulfonyl)-4-((4-(2-(tert-butyl)-5,5-dimethyl-1,3-dioxan-2-yl)benzyl)amino)-3-methoxybenzamide (0.160 g, 0.279 mmol) and triphenylphosphine (0.110 g, 0.418 mmol), were refluxed in MeOH (2.7 mL) for 3h and then cooled to room temperature. 3 drops of 2N NaOH was added to hydrolyze the aza-ylide and stirred overnight at room temperature. The reaction mixture was concentrated under reduced pressure and the residue was purified by silica gel column chromatography eluting with 0-10% MeOH/DCM to isolate a white solid (37 mg, 24%).
1H NMR (400 MHz, CDCl3) δ 7.43 (dd, J = 8.3, 1.9 Hz, 1H), 7.38 (d, J = 2.0 Hz, 1H), 7.31 (s, 4H), 6.56 (d, J = 8.4 Hz, 1H), 5.18 (t, J = 5.7 Hz, 1H), 4.40 (dd, J = 13.8, 4.3 Hz, 2H), 3.90 (s, 3H), 3.86 (d, J = 4.5 Hz, 1H), 3.64 – 3.60 (m, 1H), 3.47 (t, J = 6.0 Hz, 2H), 3.33 (s, 4H), 2.11 (dp, J = 7.7, 5.9 Hz, 2H), 1.23 (s, 3H), 0.91 (s, 9H), 0.52 (s, 3H).

NVS-STG4 (4)

N-((3-aminopropyl)sulfonyl)-4-((4-(2-(tert-butyl)-5,5-dimethyl-1,3-dioxan-2-yl)benzyl)amino)-3-methoxybenzamide (37 mg, 0.068 mmol) and 3-(3-(but-3-yn-1-yl)-3H-diazirin-3-yl)propanoic acid (14 mg, 0.084 mmol) were combined in DMF (300 µl) and Hunig's Base (40 µl, 0.229 mmol) and stirred at rt for 10 min. Then HATU (39 mg, 0.103 mmol) was added in one portion and stirred for 4h at rt. The crude reaction mixture was filtered and purified directly by preparative reverse-phase HPLC (Column: Waters X-Bridge, C18 OBD, 5 µm, 30 × 50 mm, 75 mL/min, 55-80% acetonitrile:water (both containing 0.1% formic acid) gradient over 14 min). The isolated fractions were lyophilized to afford a white solid (16.8 mg, 34%).

^{1}H NMR (400 MHz, DMSO) δ 11.56 (s, 1H), 7.96 (t, *J* = 5.8 Hz, 1H), 7.43 (dd, *J* = 8.4, 1.9 Hz, 1H), 7.40 (d, *J* = 1.9 Hz, 1H), 7.33 (d, *J* = 7.9 Hz, 2H), 7.19 (d, *J* = 7.9 Hz, 2H), 6.60 (t, *J* = 6.9 Hz, 1H), 6.50 (d, *J* = 8.5 Hz, 1H), 4.45 (d, *J* = 6.2 Hz, 2H), 3.89 (s, 3H), 3.47 (t, *J* = 8.0 Hz, 2H), 3.33 (s, 2H), 3.20 (d, *J* = 11.0 Hz, 2H), 3.11 (q, *J* = 6.4 Hz, 2H), 2.82 (t, *J* = 2.7 Hz, 1H), 1.96 (td, *J* = 7.4, 2.7 Hz, 2H), 1.84 (dd, *J* = 8.8, 6.7 Hz, 2H), 1.77 (p, *J* = 6.1 Hz, 2H), 1.61 (dd, *J* = 8.8, 6.7 Hz, 2H), 1.53 (t, *J* = 7.4 Hz, 2H), 1.15 (s, 3H), 0.84 (s, 9H), 0.47 (s, 3H).

^{1}H NMR (400 MHz, $CDCl_3$) δ 7.38 (dd, *J* = 8.3, 2.0 Hz, 1H), 7.31 (s, 5H), 7.26 (s, 1H), 6.59 (d, *J* = 8.3 Hz, 1H), 6.37 (t, *J* = 6.1 Hz, 1H), 6.02 (s, 1H), 4.44 (s, 2H), 3.92 (s, 3H), 3.66 (t, *J* = 7.3 Hz, 2H), 3.46 (q, *J* = 6.4 Hz, 2H), 3.34 (s, 4H), 2.13 (p, *J* = 6.9 Hz, 2H), 2.02 – 1.95 (m, 4H), 1.83 (dd, *J* = 8.5, 6.7 Hz, 2H), 1.67 – 1.51 (m, 2H), 1.34 (t, *J* = 7.3 Hz, 1H), 1.24 (s, 3H), 0.92 (s, 9H), 0.53 (s, 3H).

^{13}C NMR (101 MHz, $CDCl_3$) δ 173.34 (C), 165.57 (C), 146.40 (C), 143.50 (C), 137.11 (C), 135.85 (C), 130.57 (CH), 126.57 (CH), 123.18 (CH), 117.53 (C), 108.78 (CH), 108.28 (CH), 104.64 (C), 82.79 (CH), 71.65 (CH_2), 69.50 (CH), 55.93 (CH_3), 51.38 (CH_2), 47.14 (CH_2), 46.41 (CH_2), 39.89 (C), 38.08 (CH_2), 32.33 (CH_2), 30.45 (CH_2), 30.14 (C), 28.56 (CH_2), 27.95 (C), 25.09 (CH_3), 23.28 (CH_2), 23.19 (CH_3), 22.20 (CH_3), 13.33 (CH_3), 8.67 (CH).

HRMS-ESI (*m/z*) $[M+H]^+$ calculated for $C_{36}H_{50}N_5O_7S$, 696.3431; found, 696.3430.

^{1}H NMR (400 MHz, DMSO) δ 11.99 (s, 1H), 7.69 – 7.62 (m, 2H), 7.38 (d, *J* = 7.9 Hz, 2H), 7.22 (d, *J* = 8.0 Hz, 2H), 7.00 (t, *J* = 5.9 Hz, 1H), 6.68 – 6.59 (m, 2H), 4.37 (d, *J* = 5.9 Hz, 2H), 3.35 (d, *J* = 11.0 Hz, 2H), 3.22 (d, *J* = 10.8 Hz, 2H), 1.17 (s, 3H), 0.86 (s, 9H), 0.47 (s, 3H).

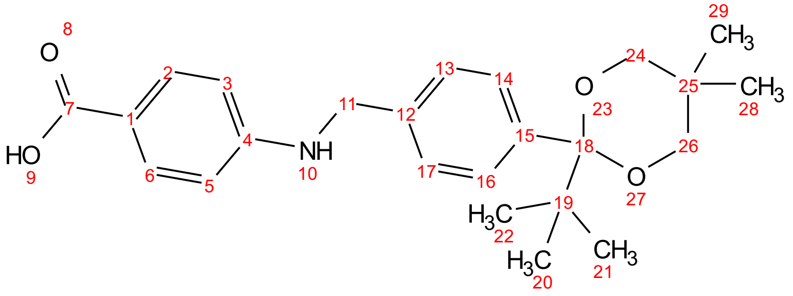

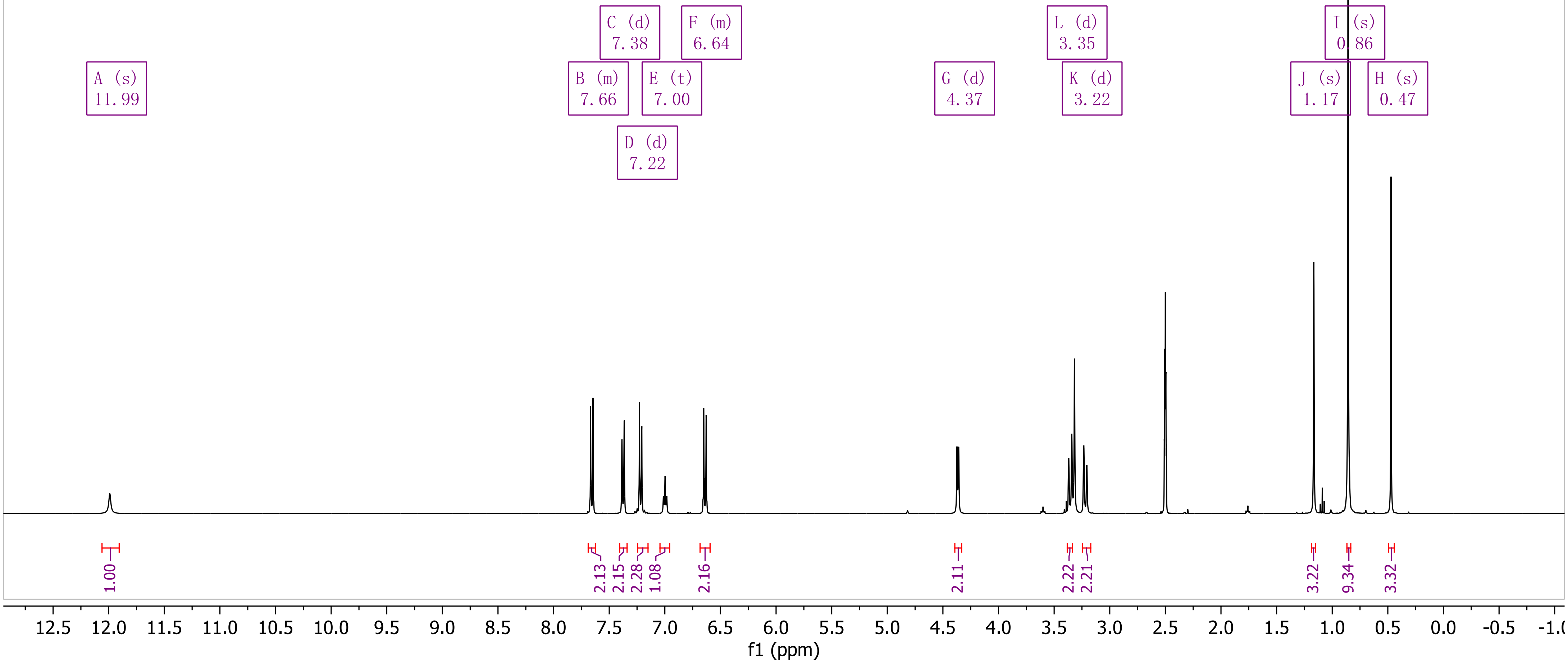

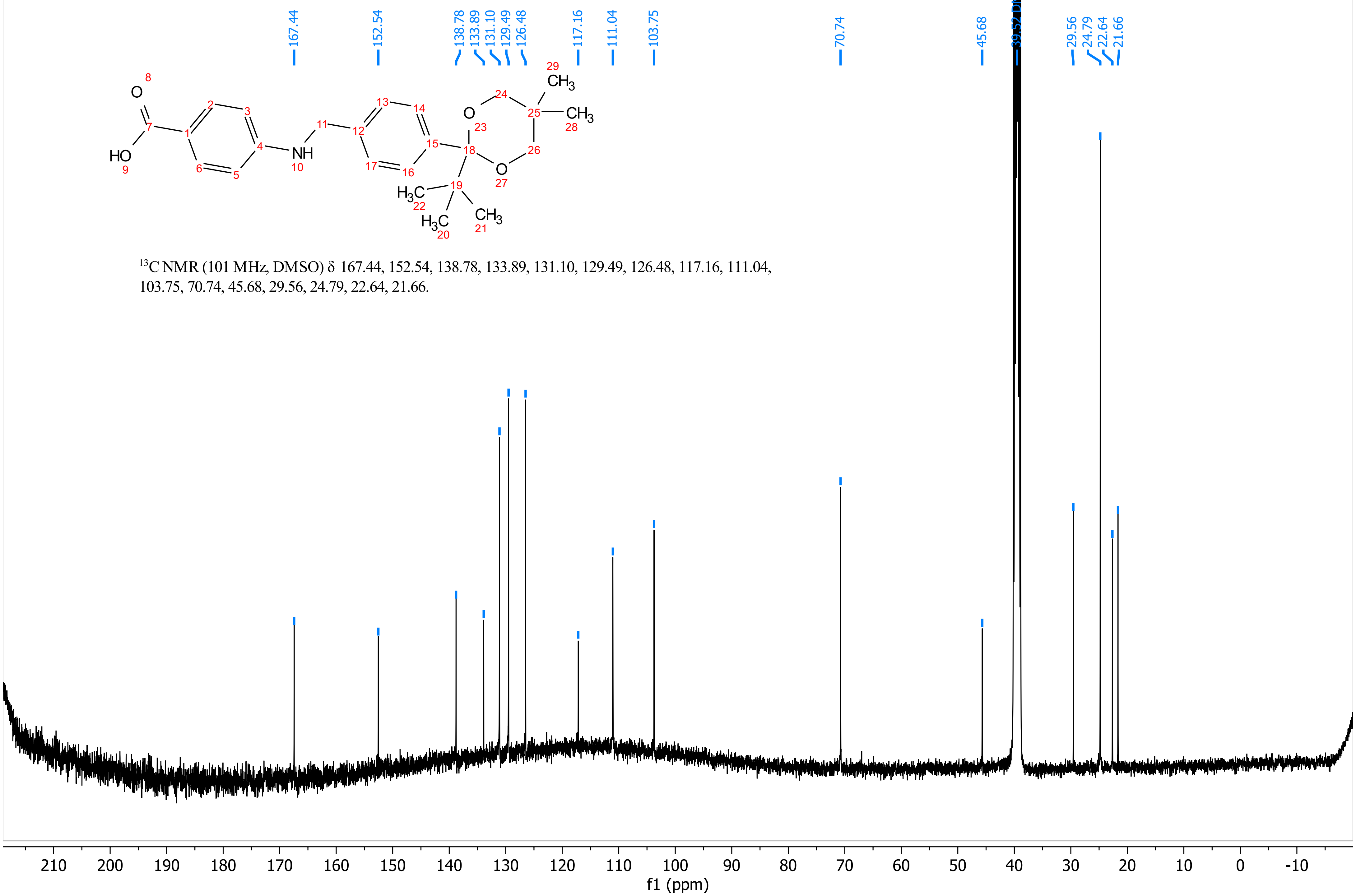

^{13}C NMR (101 MHz, DMSO) δ 167.44, 152.54, 138.78, 133.89, 131.10, 129.49, 126.48, 117.16, 111.04, 103.75, 70.74, 45.68, 29.56, 24.79, 22.64, 21.66.

^{1}H NMR (400 MHz, $CDCl_3$) δ 7.70 – 7.66 (m, 2H), 7.47 – 7.37 (m, 2H), 4.50 (s, 2H), 1.35 (s, 9H).

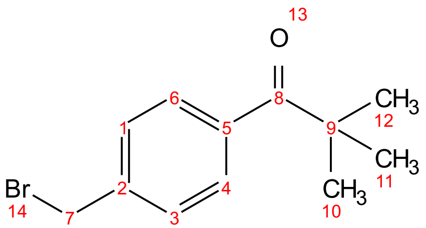

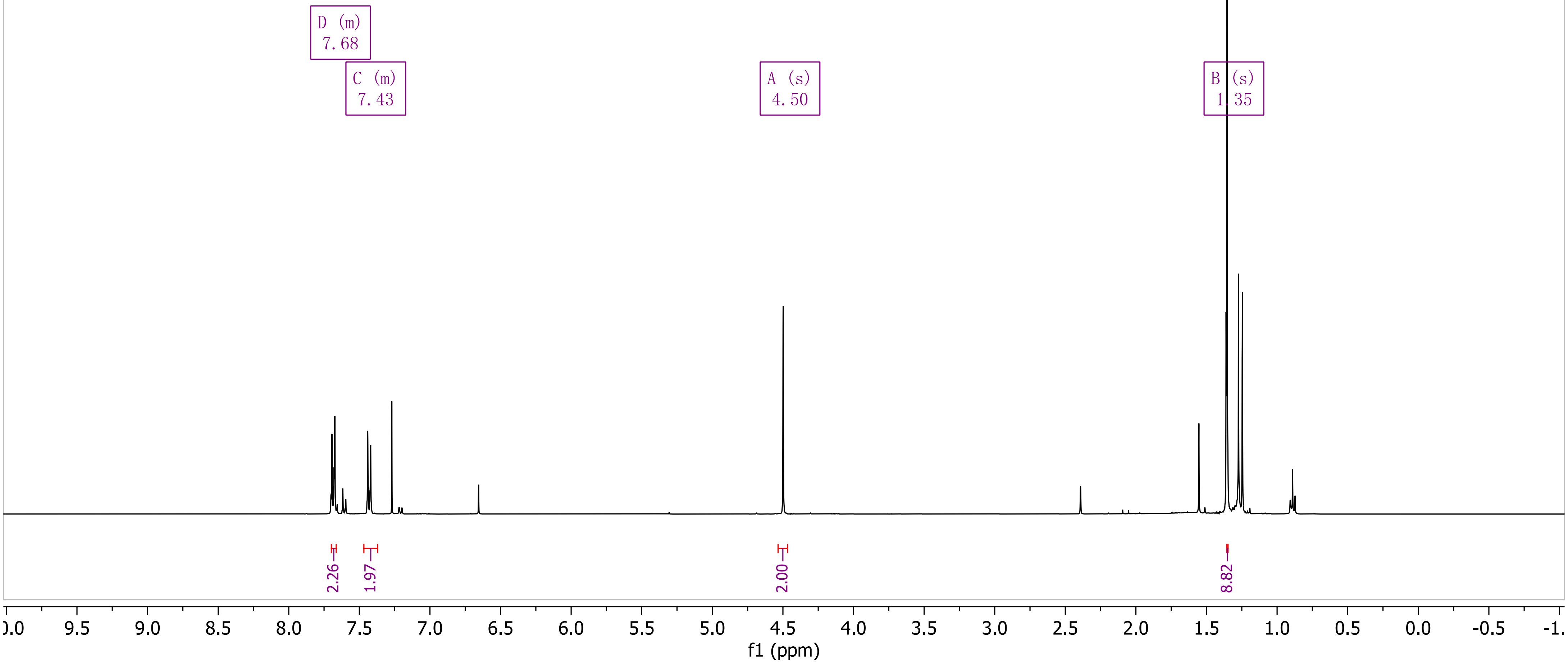

^{1}H NMR (400 MHz, $CDCl_3$) δ 7.41 – 7.37 (m, 2H), 7.33 – 7.29 (m, 2H), 4.54 (s, 2H), 3.34 (s, 4H), 1.24 (d, *J* = 4.6 Hz, 4H), 0.92 (s, 9H), 0.54 (s, 3H).

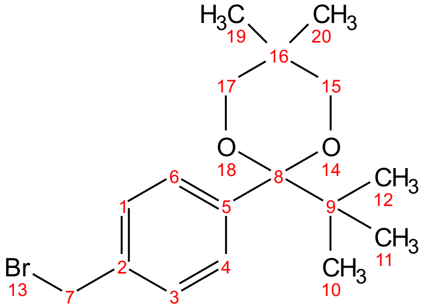

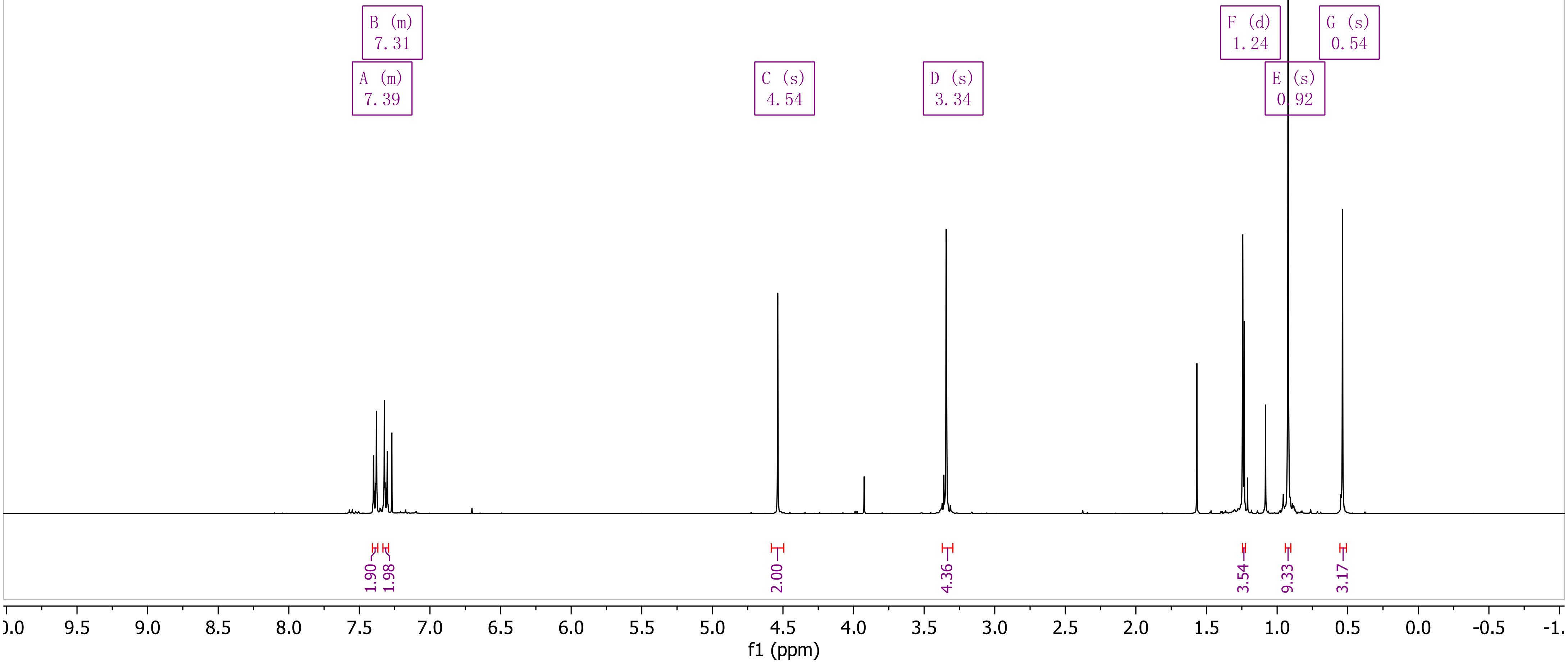

^{1}H NMR (400 MHz, DMSO) δ 7.41 (dt, J = 8.4, 1.9 Hz, 1H), 7.36 – 7.32 (m, 2H), 7.30 (t, J = 1.9 Hz, 1H), 7.22 – 7.16 (m, 2H), 6.51 (dd, J = 8.4, 1.5 Hz, 1H), 6.44 (d, J = 6.1 Hz, 1H), 4.44 (d, J = 5.9 Hz, 2H), 3.87 (d, J = 1.7 Hz, 3H), 3.74 (d, J = 1.4 Hz, 3H), 3.34 (d, J = 11.3 Hz, 2H), 3.21 (d, J = 10.7 Hz, 2H), 1.16 (s, 3H), 0.84 (d, J = 2.0 Hz, 10H), 0.46 (s, 3H).

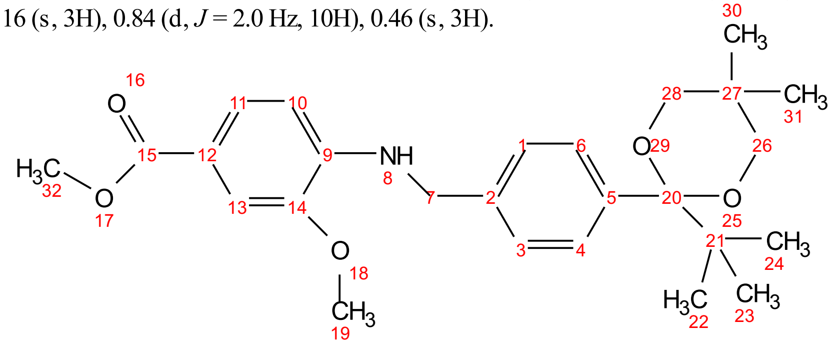

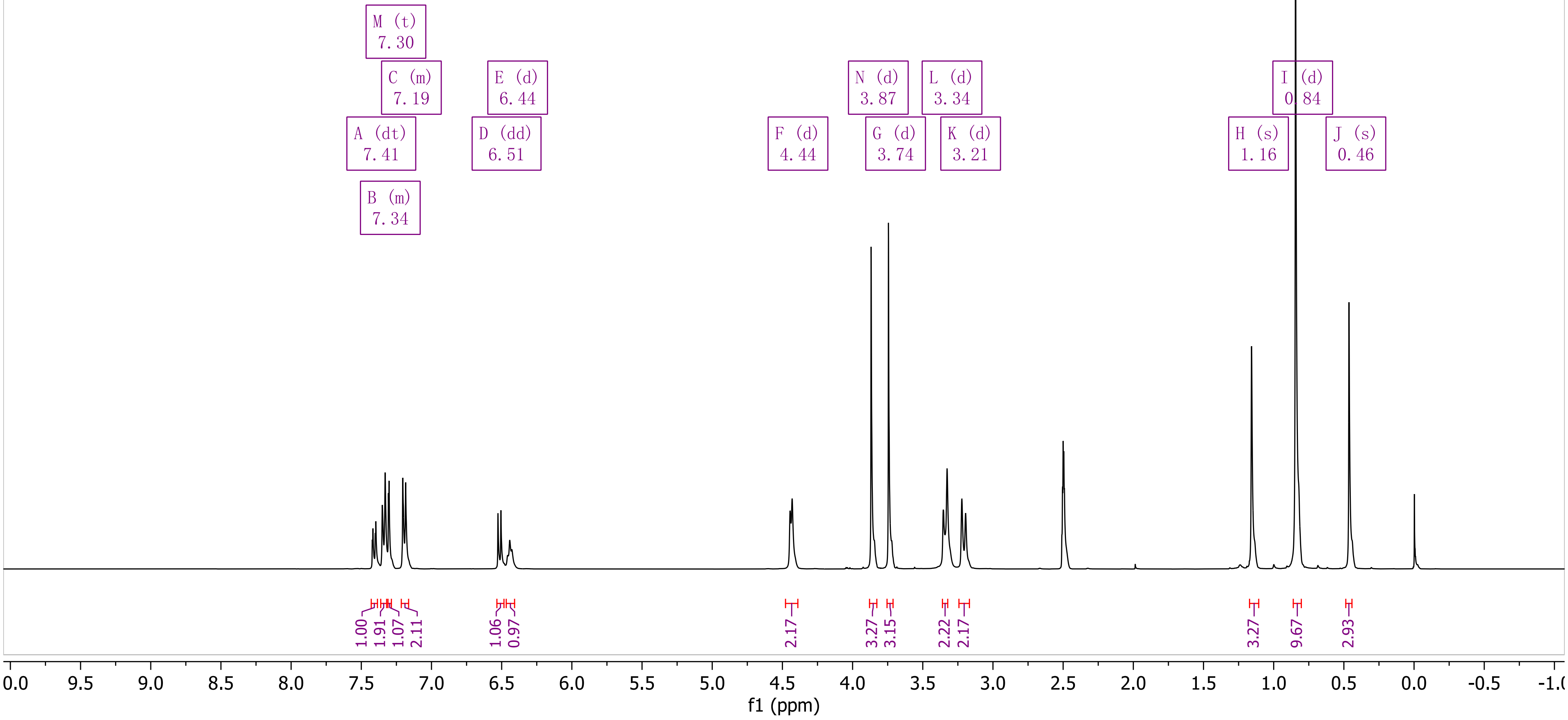

^{1}H NMR (400 MHz, DMSO) δ 7.39 – 7.33 (m, 3H), 7.28 (dd, J = 8.2, 1.6 Hz, 1H), 7.23 – 7.12 (m, 2H), 6.37 (d, J = 8.0 Hz, 1H), 5.53 (t, J = 6.2 Hz, 1H), 4.35 (d, J = 5.9 Hz, 2H), 3.80 (s, 3H), 3.33 (s, 6H), 3.23 (d, J = 10.9 Hz, 3H), 1.16 (s, 3H), 0.85 (s, 9H), 0.47 (s, 3H).

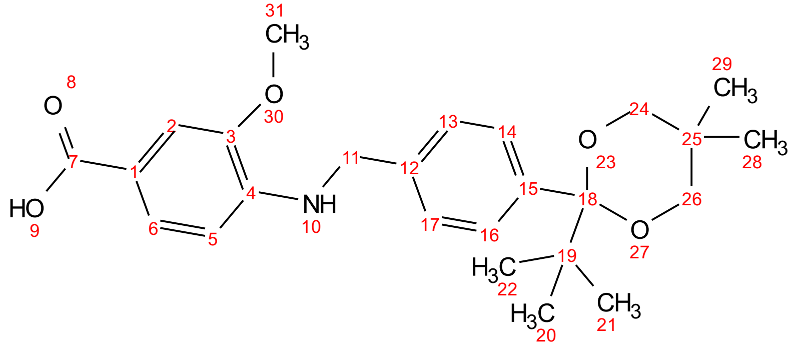

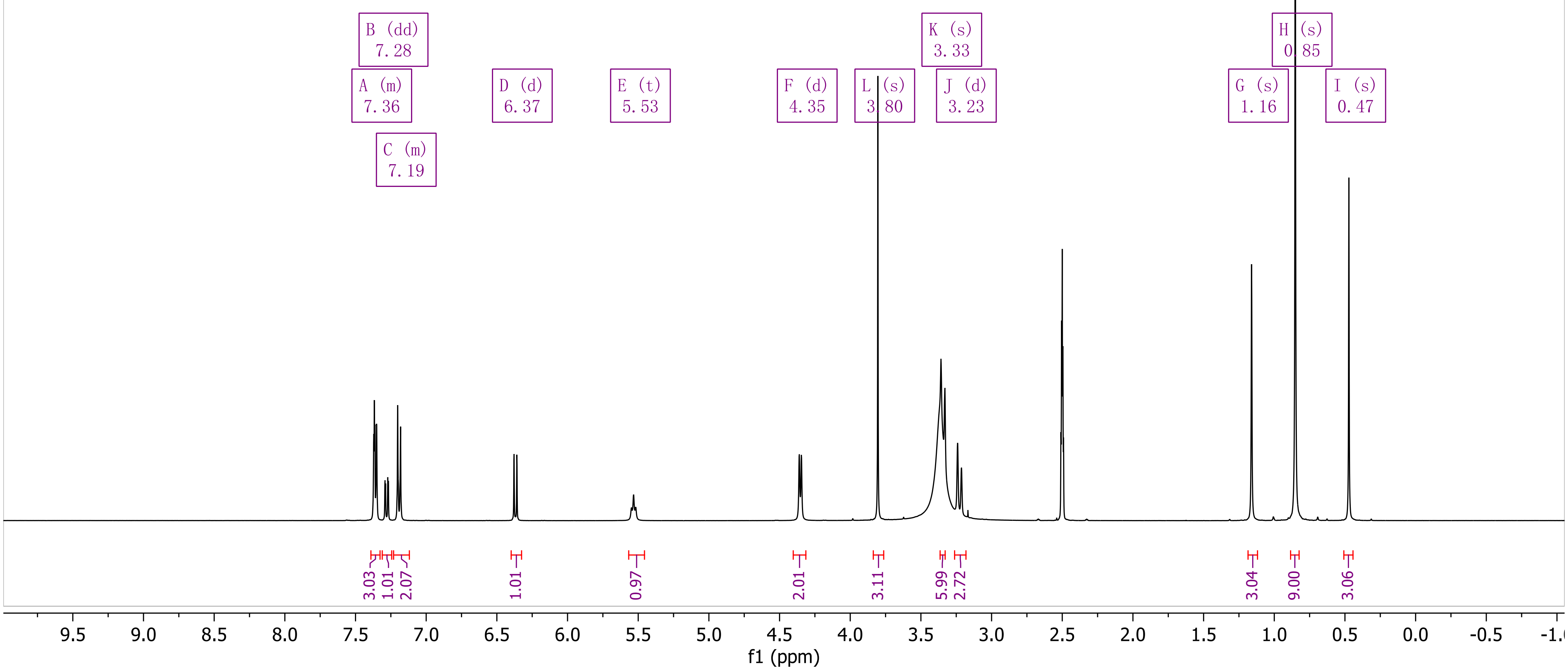

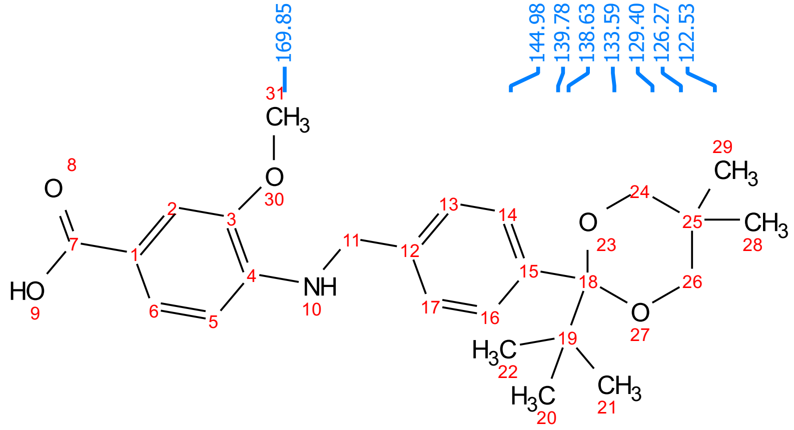

^{13}C NMR (101 MHz, DMSO) δ 169.85, 144.98, 139.78, 138.63, 133.59, 129.40, 126.27, 122.53, 110.74, 107.72, 103.77, 70.75, 55.14, 46.06, 29.57, 24.82, 22.68, 21.67.

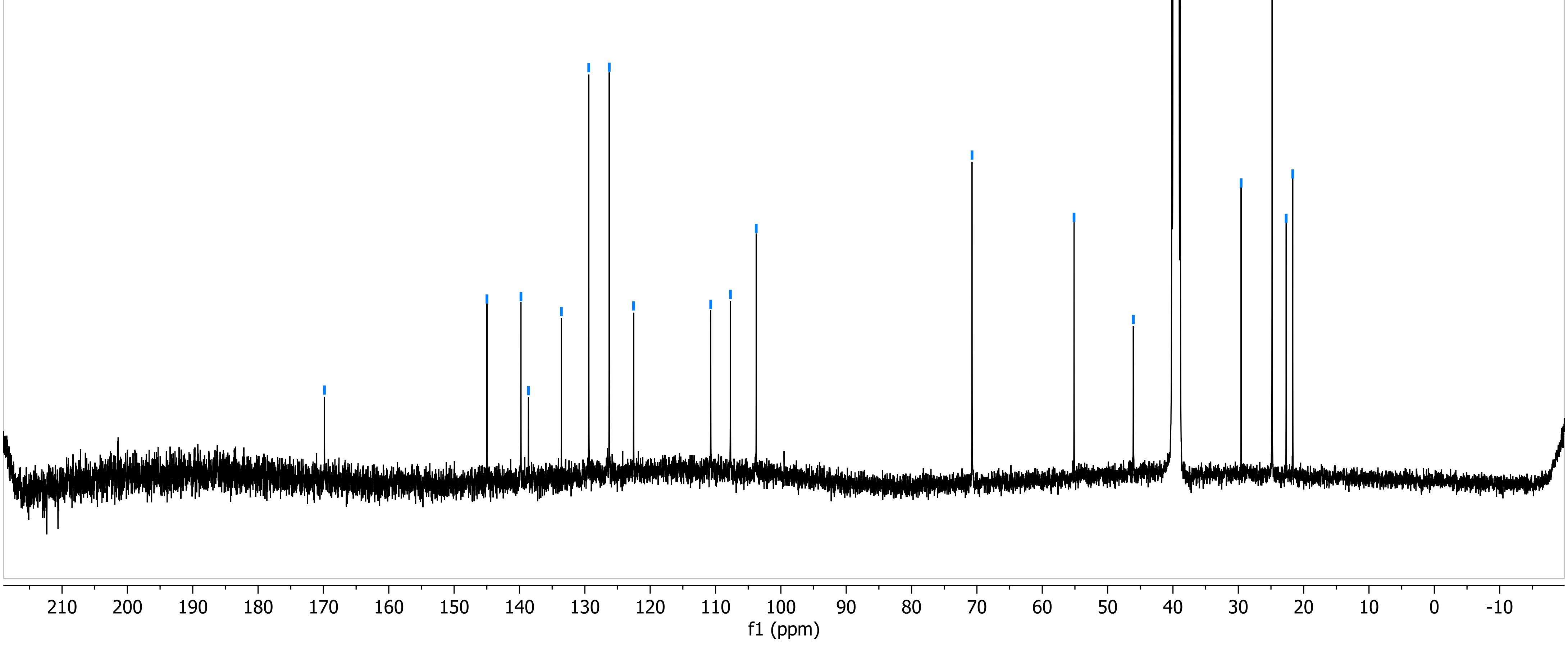

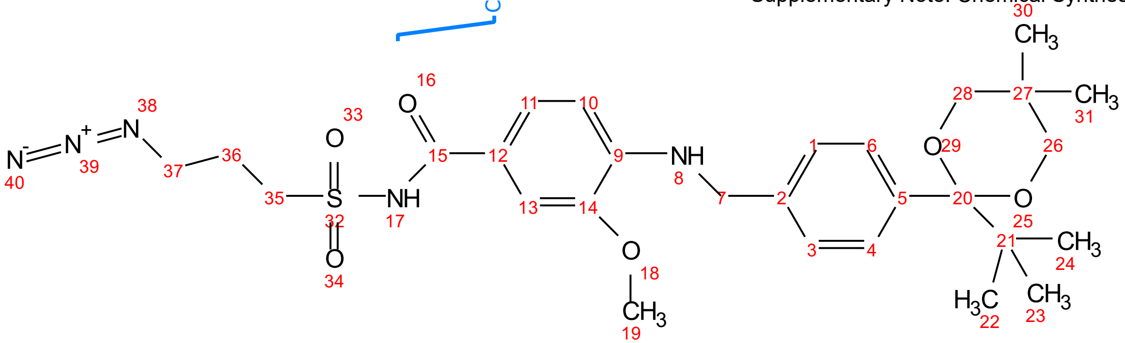

^{1}H NMR (400 MHz, $CDCl_3$) δ 7.41 (dd, *J* = 8.3, 2.0 Hz, 1H), 7.36 (d, *J* = 1.9 Hz, 1H), 7.31 (s, 4H), 6.58 (d, *J* = 8.4 Hz, 1H), 5.23 (t, *J* = 5.6 Hz, 1H), 4.43 (d, *J* = 5.2 Hz, 2H), 3.92 (s, 3H), 3.69 – 3.60 (m, 2H), 3.48 (d, *J* = 6.6 Hz, 4H), 3.26 – 3.18 (m, 2H), 2.20 – 1.98 (m, 2H), 1.23 (s, 3H), 0.92 (s, 9H), 0.52 (s, 3H).

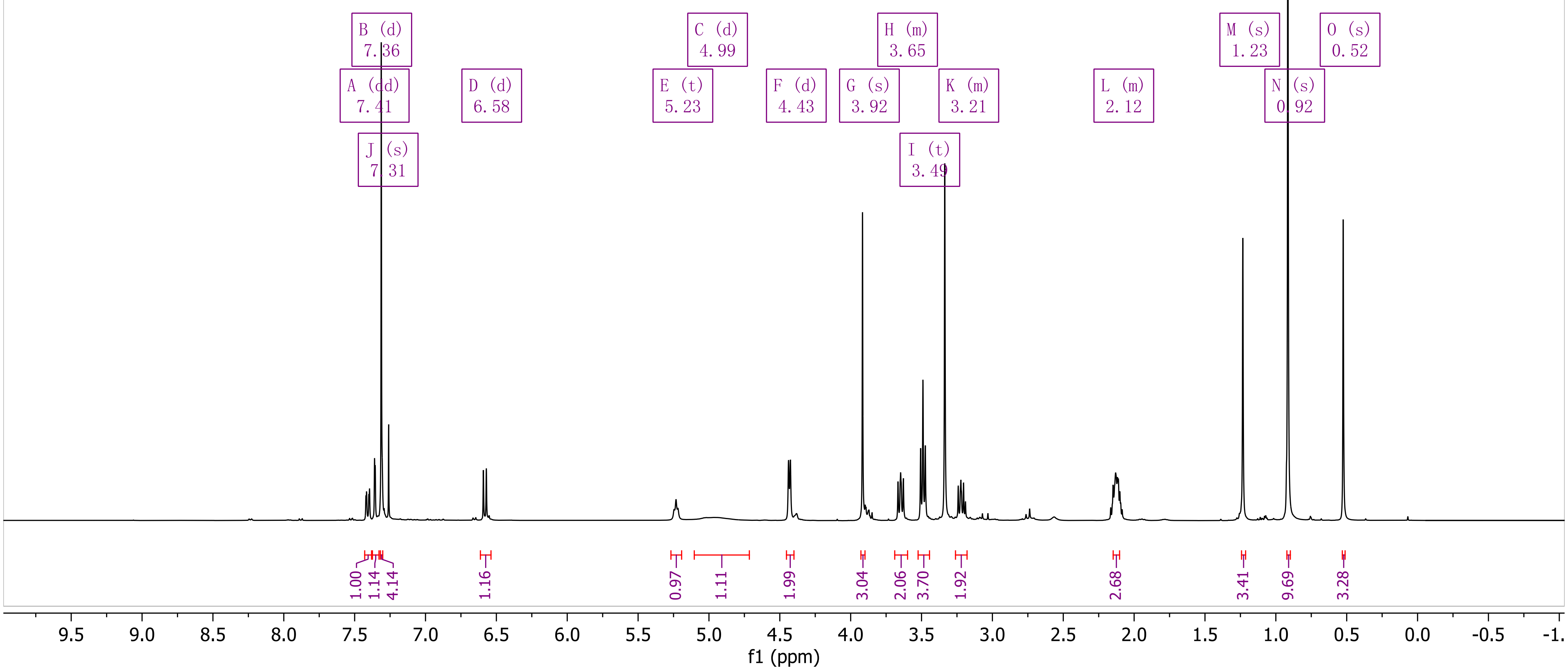

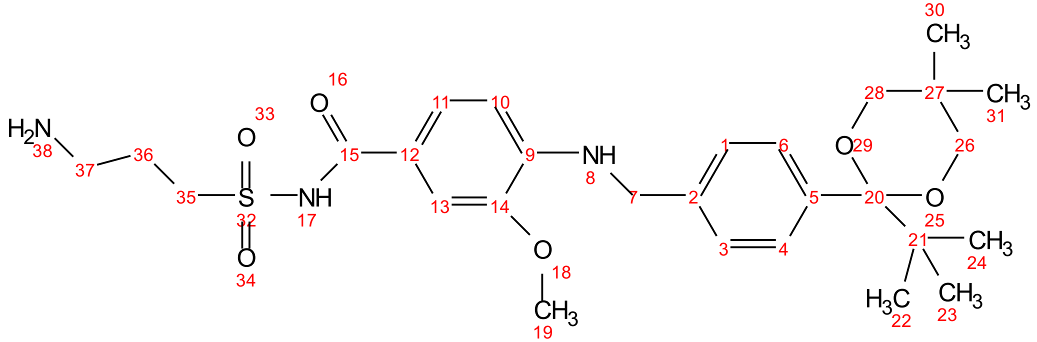

^{1}H NMR (400 MHz, $CDCl_3$) δ 7.43 (dd, J = 8.3, 1.9 Hz, 1H), 7.38 (d, J = 2.0 Hz, 1H), 7.31 (s, 4H), 6.56 (d, J = 8.4 Hz, 1H), 5.18 (t, J = 5.7 Hz, 1H), 4.40 (dd, J = 13.8, 4.3 Hz, 2H), 3.90 (s, 3H), 3.86 (d, J = 4.5 Hz, 1H), 3.64 – 3.60 (m, 1H), 3.47 (t, J = 6.0 Hz, 2H), 3.33 (s, 4H), 2.11 (dp, J = 7.7, 5.9 Hz, 2H), 1.23 (s, 3H), 0.91 (s, 9H), 0.52 (s, 3H).

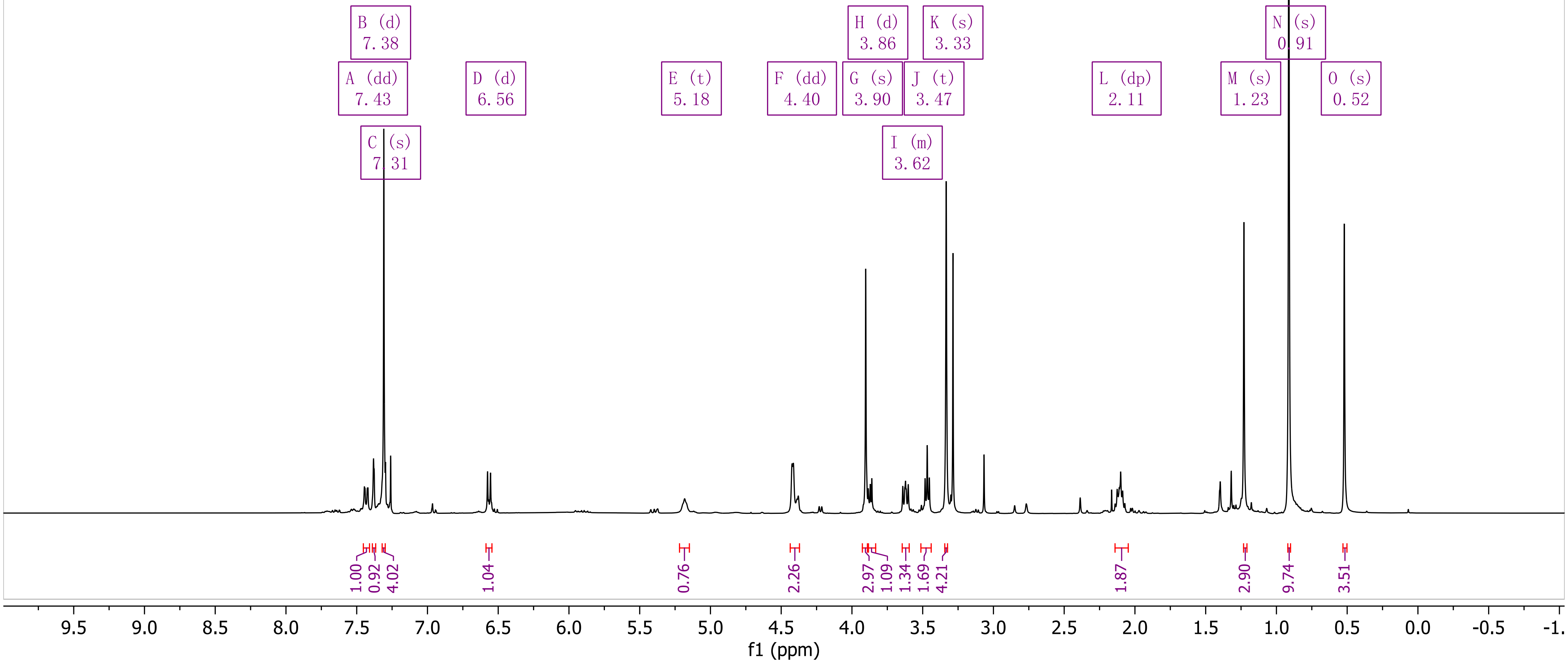

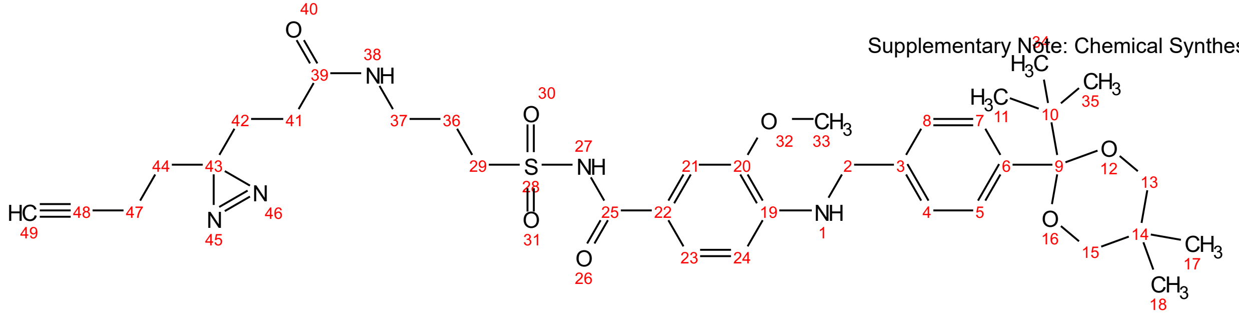

^{1}H NMR (400 MHz, DMSO) δ 11.56 (s, 1H), 7.96 (t, J = 5.8 Hz, 1H), 7.43 (dd, J = 8.4, 1.9 Hz, 1H), 7.40 (d, J = 1.9 Hz, 1H), 7.33 (d, J = 7.9 Hz, 2H), 7.19 (d, J = 7.9 Hz, 2H), 6.60 (t, J = 6.9 Hz, 1H), 6.50 (d, J = 8.5 Hz, 1H), 4.45 (d, J = 6.2 Hz, 2H), 3.89 (s, 3H), 3.47 (t, J = 8.0 Hz, 2H), 3.33 (s, 2H), 3.20 (d, J = 11.0 Hz, 2H), 3.11 (q, J = 6.4 Hz, 2H), 2.82 (t, J = 2.7 Hz, 1H), 1.96 (td, J = 7.4, 2.7 Hz, 2H), 1.84 (dd, J = 8.8, 6.7 Hz, 2H), 1.77 (p, J = 6.1 Hz, 2H), 1.61 (dd, J = 8.8, 6.7 Hz, 2H), 1.53 (t, J = 7.4 Hz, 2H), 1.15 (s, 3H), 0.84 (s, 9H), 0.47 (s, 3H).

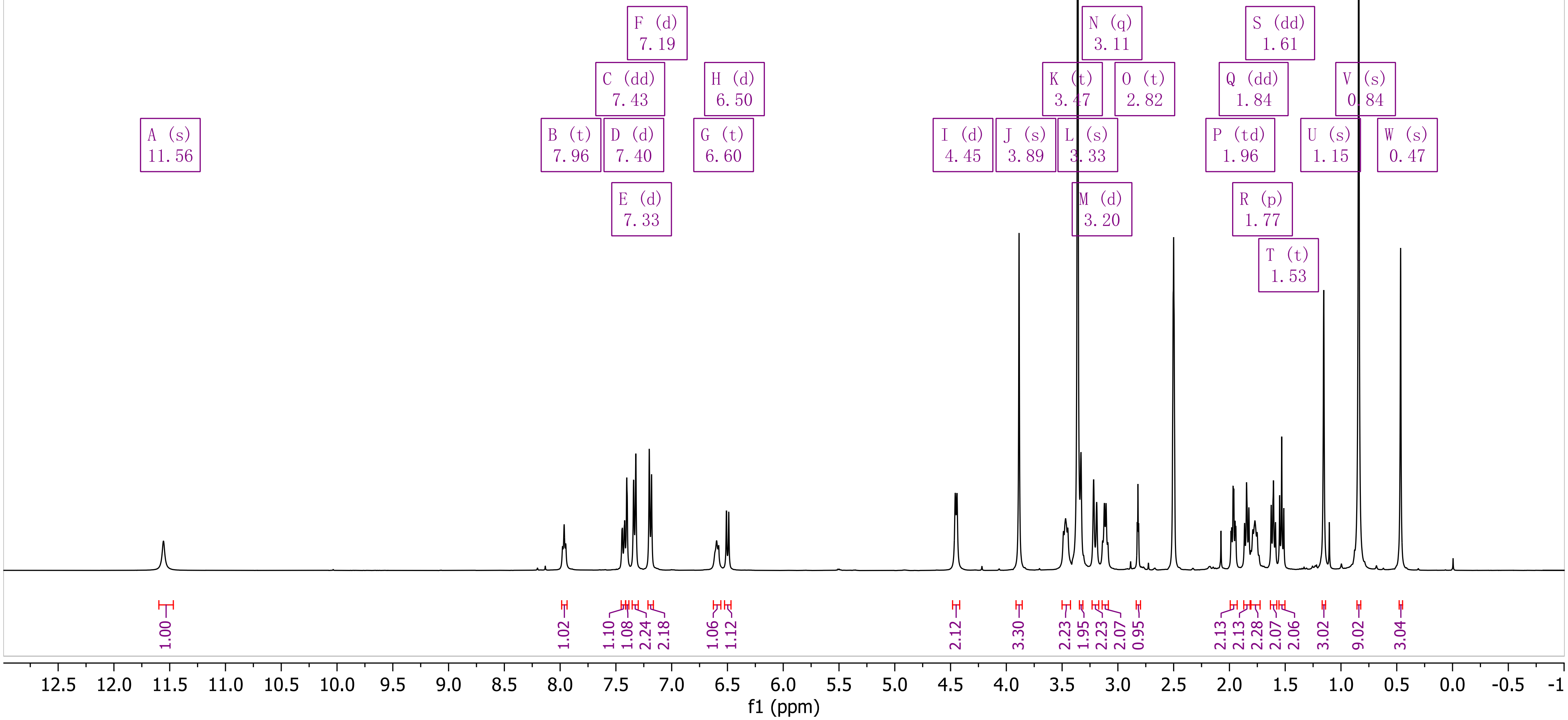

Supplementary Note: Chemical Synthesis

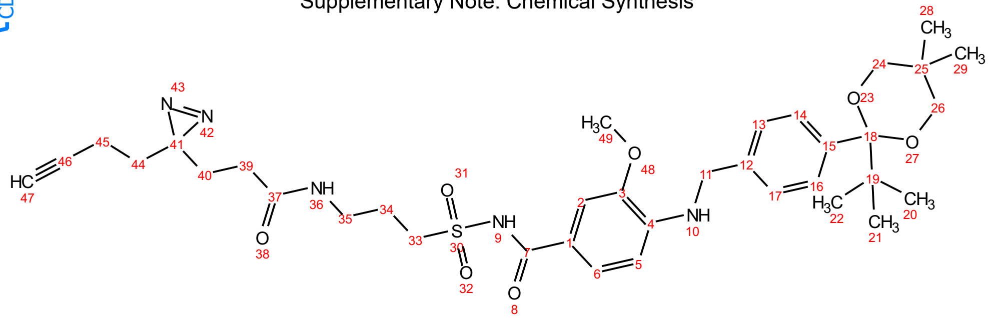

^{1}H NMR (400 MHz, $CDCl_3$) δ 7.38 (dd, *J* = 8.3, 2.0 Hz, 1H), 7.31 (s, 5H), 7.26 (s, 1H), 6.59 (d, *J* = 8.3 Hz, 1H), 6.37 (t, *J* = 6.1 Hz, 1H), 6.02 (s, 1H), 4.44 (s, 2H), 3.92 (s, 3H), 3.66 (t, *J* = 7.3 Hz, 2H), 3.46 (q, *J* = 6.4 Hz, 2H), 3.34 (s, 4H), 2.13 (p, *J* = 6.9 Hz, 2H), 2.02 – 1.95 (m, 4H), 1.83 (dd, *J* = 8.5, 6.7 Hz, 2H), 1.67 – 1.51 (m, 2H), 1.34 (t, *J* = 7.3 Hz, 1H), 1.24 (s, 3H), 0.92 (s, 9H), 0.53 (s, 3H).

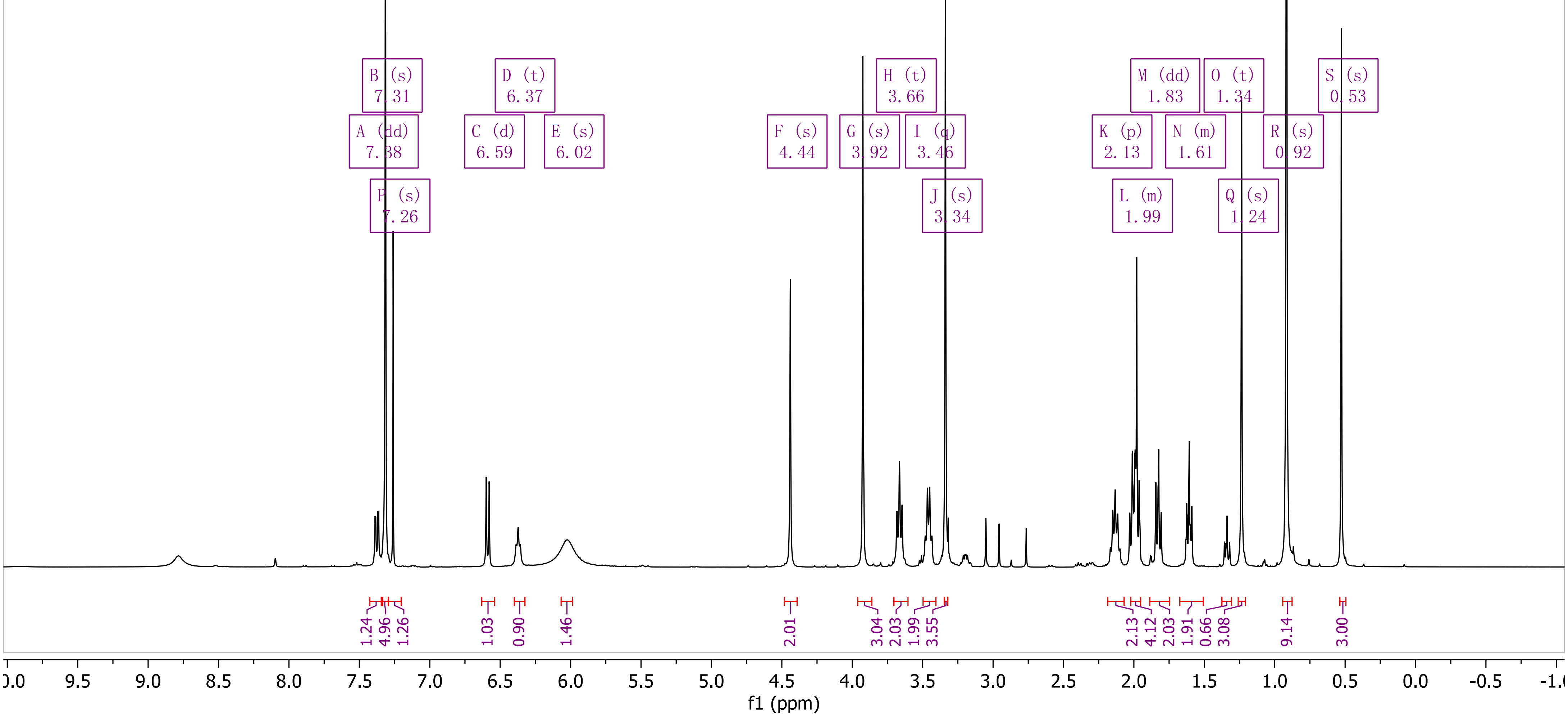

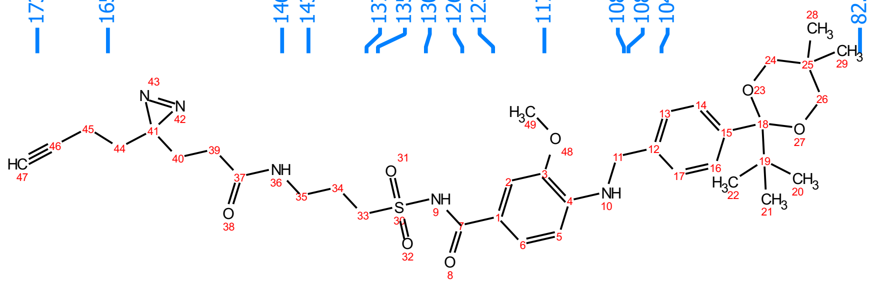

^{13}C NMR (101 MHz, $CDCl_3$) δ 173.34, 165.57, 146.40, 143.50, 137.11, 135.85, 130.57, 126.57, 123.18, 117.53, 108.78, 108.28, 104.64, 82.79, 71.65, 69.50, 55.93, 51.38, 47.14, 46.41, 39.89, 38.08, 32.33, 30.45, 30.14, 28.56, 27.95, 25.09, 23.28, 23.19, 22.20, 13.33, 8.67.

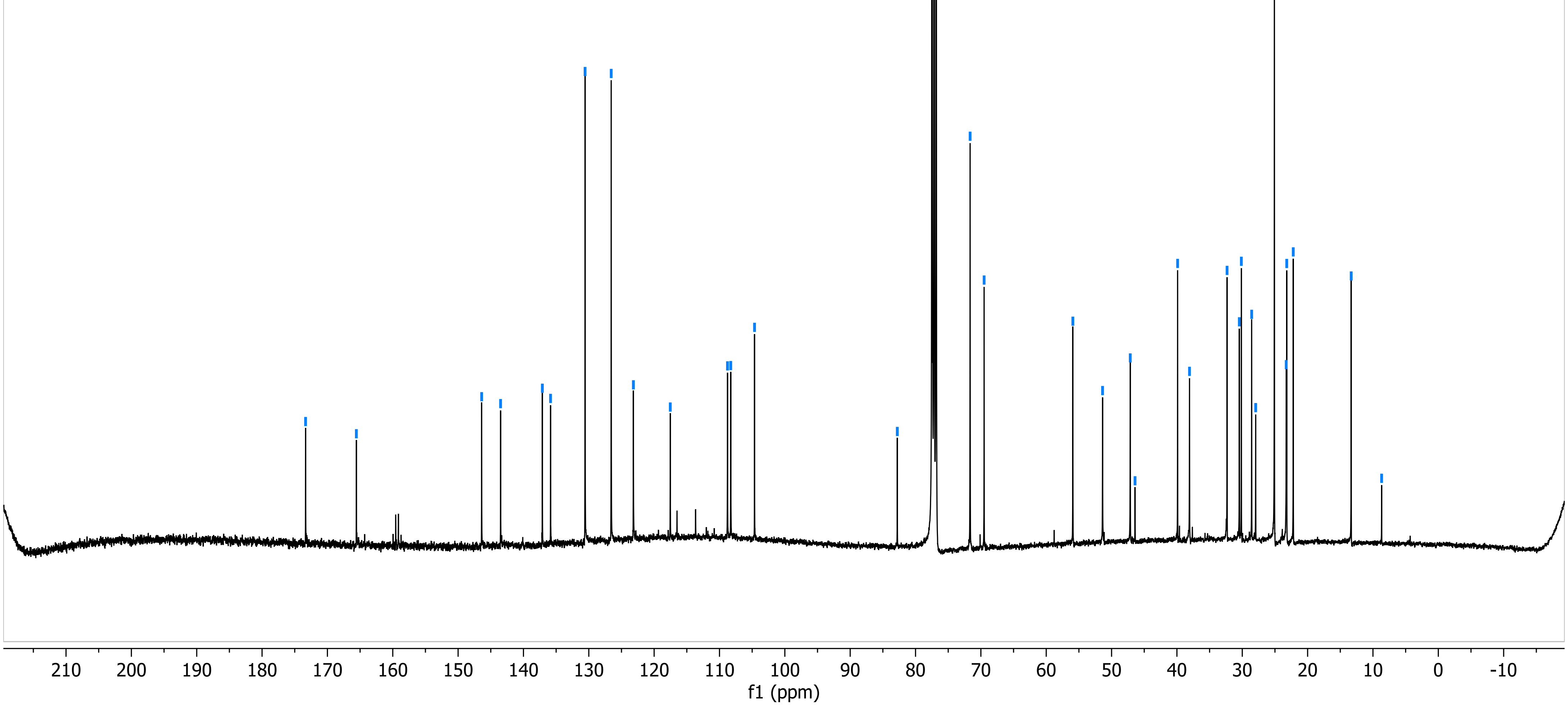

Supplementary Note: Chemical Synthesis

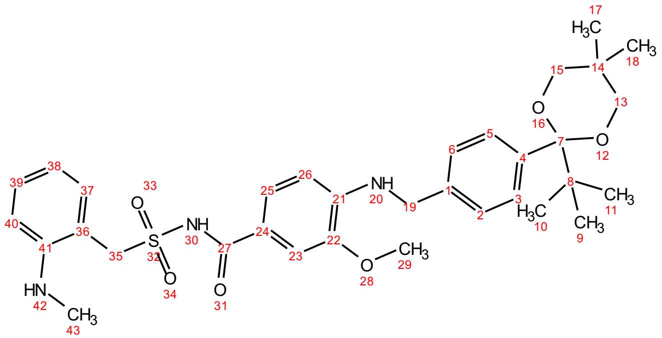

^{1}H NMR (400 MHz, $CDCl_3$) δ 7.42 – 7.36 (m, 1H), 7.35 – 7.29 (m, 3H), 7.24 (d, J = 4.0 Hz, 5H), 6.48 (d, J = 8.3 Hz, 1H), 4.94 (s, 2H), 4.34 (s, 2H), 3.79 (s, 3H), 3.26 (s, 4H), 3.05 (s, 3H), 2.68 (s, 1H), 1.15 (s, 3H), 0.84 (s, 9H), 0.44 (s, 3H).

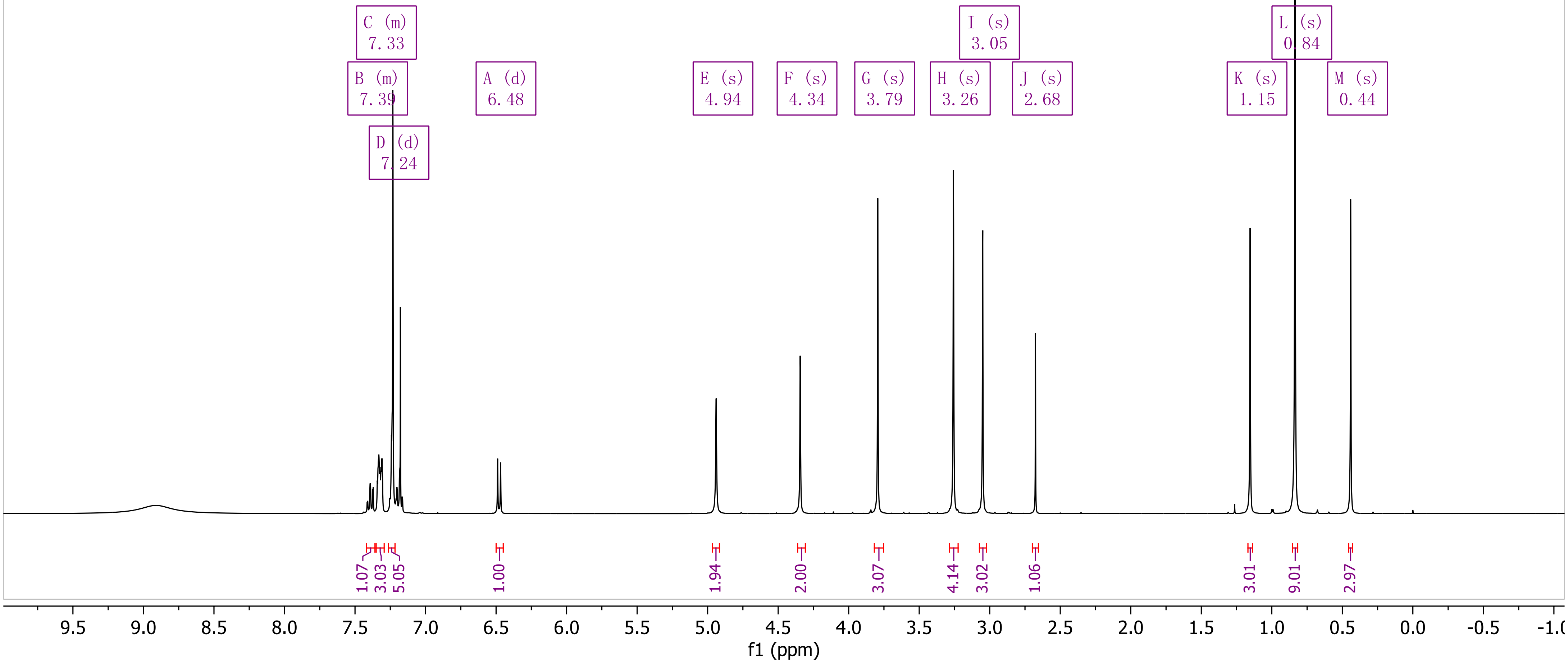

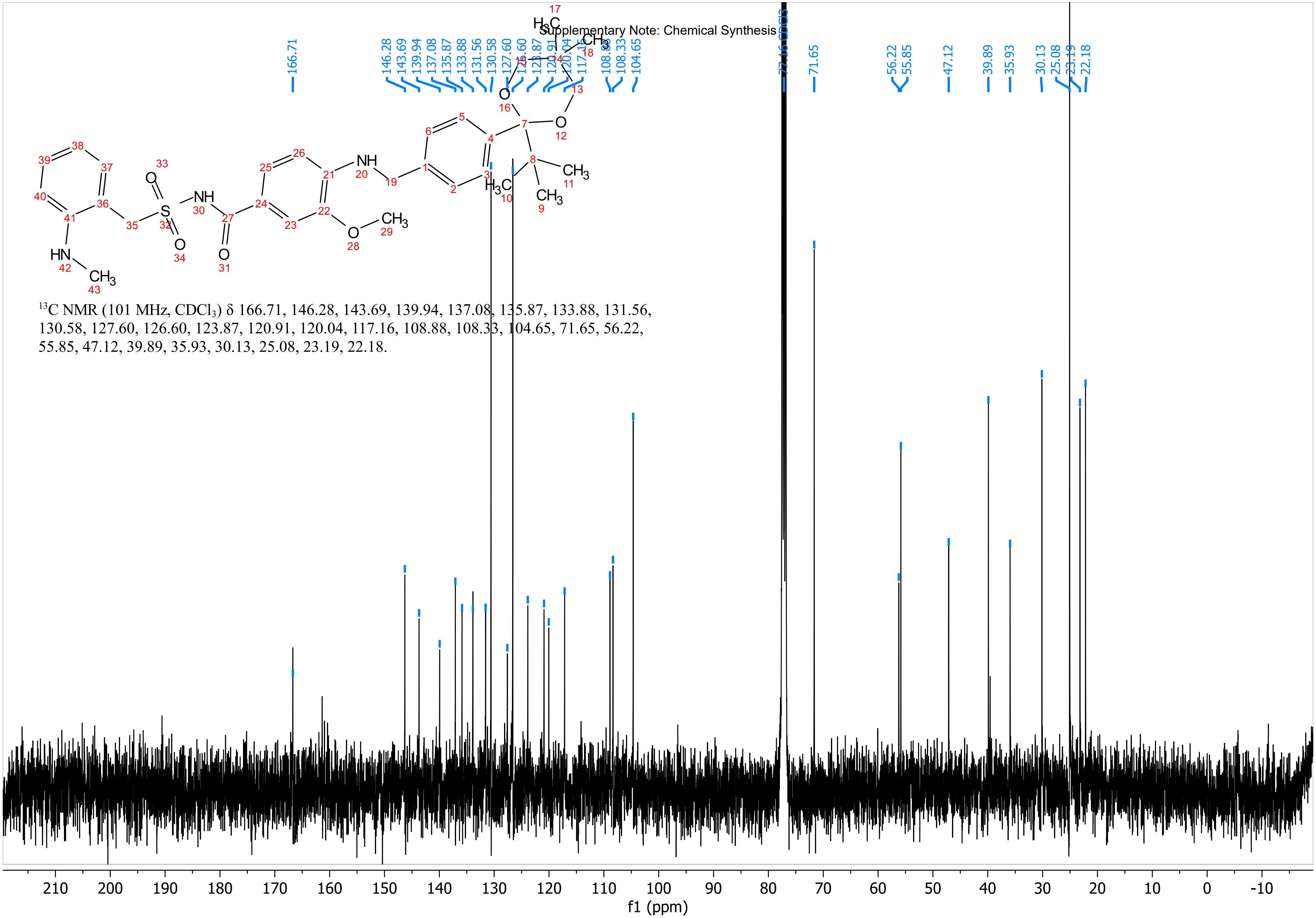
166.71
146.28
143.69
139.94
137.08
135.87
133.88
131.56
130.58
127.60
126.60
123.87
120.91
120.04
117.16
108.88
108.33
104.65
77.16 CDCl3
71.65
56.22
55.85
47.12
39.89
35.93
30.13
25.08
23.19
22.18
^{13}C NMR (101 MHz, CDCl3) δ 166.71, 146.28, 143.69, 139.94, 137.08, 135.87, 133.88, 131.56, 130.58, 127.60, 126.60, 123.87, 120.91, 120.04, 117.16, 108.88, 108.33, 104.65, 71.65, 56.22, 55.85, 47.12, 39.89, 35.93, 30.13, 25.08, 23.19, 22.18.
f1 (ppm)

Supplementary Table 1. Cryo-EM data collection and structure refinement statistics.

Structure	STING+cGAMP+STG2	STING+cGAMP+STG2+C53
Magnification	81,000	81,000
Voltage (kV)	300	300
Electron exposure (e^{-}/Å^2)	60	60
Defocus range (µm)	1.5-2.5	1.5-2.5
Pixel size (Å)	1.08	1.08
Symmetry imposed	C2	C2
Initial particle images (no.)	1,568,146	3,207,170
Final particle images (no.)	117,249	292,483
Map resolution (Å)	4.0	2.9
FSC threshold	0.143	0.143
Initial model used (PDB code)	7SII	7SII
Model resolution (Å)	4.0	3.0
FSC threshold	0.5	0.5
Map sharpening B factor (Å^2)	-200	-40
Model Composition		
Non-hydrogen atoms	10,232	14,740
Protein residues	1276	1,272
Ligands	2 cGAMP<br>2 C53	2 cGAMP<br>2 STG2<br>2 C53
Protein B factors (Å^2)	113.2	69.9
Ligand B factors (Å^2)	102.5	51.8
R.m.s. deviations		
Bond length (Å)	0.004	0.003
Bond angle (°)	0.729	0.68
Validation		
Molprobity score	1.83	1.41
Clashscore	10.22	5.1
Poor rotamers (%)	0	0.94
Ramachandran plot		
Favored (%)	95.7	97.3
Allowed (%)	4.3	2.7
Outliers (%)	0	0
