# Supplementary material for: Activation of human STING by a molecular glue-like compound
Source: Nat Chem Biol. 2023 Oct 12;20(3):365–72. doi: 10.1038/s41589-023-01434-y (PMC10907298; doi:10.1038/s41589-023-01434-y)
Supplement: Supplementary file 2 — Reporting Summary [file 41589_2023_1434_MOESM2_ESM.pdf]

nature portfolio

Corresponding author(s): Steve Canham, Xuewu Zhang, Xiaochen Bai, Yan Feng

Last updated by author(s): Aug 17, 2023

Reporting Summary

Nature Portfolio wishes to improve the reproducibility of the work that we publish. This form provides structure for consistency and transparency in reporting. For further information on Nature Portfolio policies, see our Editorial Policies and the Editorial Policy Checklist.

Statistics

For all statistical analyses, confirm that the following items are present in the figure legend, table legend, main text, or Methods section.

n/a	Confirmed	
☐	☒	The exact sample size (*n*) for each experimental group/condition, given as a discrete number and unit of measurement
☐	☒	A statement on whether measurements were taken from distinct samples or whether the same sample was measured repeatedly
☐	☒	The statistical test(s) used AND whether they are one- or two-sided<br>*Only common tests should be described solely by name; describe more complex techniques in the Methods section.*
☒	☐	A description of all covariates tested
☒	☐	A description of any assumptions or corrections, such as tests of normality and adjustment for multiple comparisons
☐	☒	A full description of the statistical parameters including central tendency (e.g. means) or other basic estimates (e.g. regression coefficient) AND variation (e.g. standard deviation) or associated estimates of uncertainty (e.g. confidence intervals)
☐	☒	For null hypothesis testing, the test statistic (e.g. *F*, *t*, *r*) with confidence intervals, effect sizes, degrees of freedom and *P* value noted<br>*Give P values as exact values whenever suitable.*
☒	☐	For Bayesian analysis, information on the choice of priors and Markov chain Monte Carlo settings
☒	☐	For hierarchical and complex designs, identification of the appropriate level for tests and full reporting of outcomes
☒	☐	Estimates of effect sizes (e.g. Cohen's *d*, Pearson's *r*), indicating how they were calculated

Our web collection on statistics for biologists contains articles on many of the points above.

Software and code

Policy information about availability of computer code

Data collection	Excel v16.75<br>Motioncorr2 program v1.2<br>RELION 3.1
Data analysis	Graphpad Prism v9.4.1<br>TIBCO Spotfire 11.4.2<br>ImmunoSpot software v3.2<br>Chimera 1.16<br>Phenix 1.18<br>Coot 0.98

For manuscripts utilizing custom algorithms or software that are central to the research but not yet described in published literature, software must be made available to editors and reviewers. We strongly encourage code deposition in a community repository (e.g. GitHub). See the Nature Portfolio guidelines for submitting code & software for further information.

Data

Policy information about availability of data

All manuscripts must include a data availability statement. This statement should provide the following information, where applicable:

- Accession codes, unique identifiers, or web links for publicly available datasets
- A description of any restrictions on data availability
- For clinical datasets or third party data, please ensure that the statement adheres to our policy

The atomic coordinates and the cryo-EM maps of STING bound to cGAMP/NVS-STG2 have been deposited to the RCSB PDB (PDB IDs: 8FLK) and the electron microscopy data bank (EMDB) (EMBD ID: EMD-29281) respectively. The coordinates and map of STING bound ot cGAMP/NVS-STG2/C53 have been deposited to the PDB and EMDB with accession IDs of 8FLM and EMD-29282, respectively. Additional data can be provided upon written request to the corresponding authors.

Human research participants

Policy information about studies involving human research participants and Sex and Gender in Research.

Reporting on sex and gender	N/A
Population characteristics	N/A
Recruitment	N/A
Ethics oversight	N/A

Note that full information on the approval of the study protocol must also be provided in the manuscript.

Field-specific reporting

Please select the one below that is the best fit for your research. If you are not sure, read the appropriate sections before making your selection.

☒ Life sciences ☐ Behavioural & social sciences ☐ Ecological, evolutionary & environmental sciences

For a reference copy of the document with all sections, see nature.com/documents/nr-reporting-summary-flat.pdf

Life sciences study design

All studies must disclose on these points even when the disclosure is negative.

Sample size

For in vivo testing STING agonists, the minimum group size of 8 mice was selected for evaluating dose related. experiments proposed in this protocol are based on studies performed in Chemical Biology & Therapeutics Department (CBT) in vivo pharmacology group and Novartis Oncology Pharmacology group who have extensive experience (>10 years) in evaluating drug candidates for efficacy in mice bearing human or murine tumors. Novartis statisticians were consulted during development of the in vivo model systems. The minimum group size of 8 was selected for evaluating dose related activity. Power Analyses were performed in SYSTAT13 using One-way ANOVA. A comparison of in-house data based on N=2 groups was used to extrapolate numbers needed for studies with N=3-4 groups.
Group Size Calculations:
Anticipated Values (mm3):
Mean Standard Deviation
Group 1 1746 342
Group 2 1134 391
Difference in means= 35%
Sample Size Needed Per Group:
N=8 for Power = 0.9 with Alpha=0.05
N=6 for Power = 0.8 with Alpha=0.05

Data exclusions

No data was excluded.

Replication

We did not replicate the study using the same conditions but the compounds have been repetitively tested in different studies under slightly different conditions to confirm the effects.

Randomization

Animals were randomized between groupes based on tumor size, so the average tumor sizes at the time of initial compound dosing was similar.

Blinding

No histology in this study therefore no blinding was used.

Reporting for specific materials, systems and methods

We require information from authors about some types of materials, experimental systems and methods used in many studies. Here, indicate whether each material, system or method listed is relevant to your study. If you are not sure if a list item applies to your research, read the appropriate section before selecting a response.

Materials & experimental systems

n/a	Involved in the study	
☐	☒	Antibodies
☐	☒	Eukaryotic cell lines
☒	☐	Palaeontology and archaeology
☐	☒	Animals and other organisms
☒	☐	Clinical data
☒	☐	Dual use research of concern

Methods

n/a	Involved in the study	
☒	☐	ChIP-seq
☒	☐	Flow cytometry
☒	☐	MRI-based neuroimaging

Antibodies

Antibodies used	pIRF3 rabbit antibody (Ser396, Cell Signaling Technology (4D4G) cat#4947)<br>HRP-Goat anti-Rabbit IgG (H+L) secondary antibody (BIO-RAD, cat#1706515)<br>R&D systems mouse IFN-γ kit (cat#EL485)
Validation	Validation by commercial suppliers of the antibodies can be found on supplier's website.

Eukaryotic cell lines

Policy information about cell lines and Sex and Gender in Research

Cell line source(s)	ATCC : THP1 (cat# TIB-202), HEK293T (cat# CRL-3216), sf9 (cat# CRL-1711)<br>Invivogen: THP1-Dual (cat# thpd-nfis)<br>ThermoFisher: FreeStyle 293-F (cat#R79007)<br>MC38 cells : Were acquired from the laboratories of James Hodge, PhD and Jeffery Schlom, PhD (National Cancer Institute/NIH).<br>The murine melanoma derived B16-SIY cells (referred to as B16-SIY; engineered to express the model SIYRYYGL (SIY) antigen to enable immune monitoring) were acquired from the Gajewski lab (University of Chicago).
Authentication	All cell lines were used as is from the suppliers and were not authenticated before use.
Mycoplasma contamination	All cell lines were routinely tested for mycoplasma contamination and tested negative prior to use.
Commonly misidentified lines<br>(See ICLAC register)	No commonly misidentified lines were used in this study.

Animals and other research organisms

Policy information about studies involving animals; ARRIVE guidelines recommended for reporting animal research, and Sex and Gender in Research

Laboratory animals	C57BL/6 WT (strain code:0000664) and human STING Knock in (10–12 weeks old, 20–25 g) were purchased from Jackson lab or bred in house. All mice were housed in state-of-the-art Association for the Assessment and Accreditation for Laboratory Animal Care (AACLAC)-accredited animal research facility at Novartis (Cambridge, MA USA) in 12H light/dark cycle at 70.6F and 58% humidity.
Wild animals	No wild animal was used in this study.
Reporting on sex	Only female mice were used in the study based on availability. We do not believe there to be a sex difference in response but further experiments would be needed to confirm this hypothesis.
Field-collected samples	No field-collected samples were used in this study.
Ethics oversight	All animal experiments procedures were performed according to the guidelines approved by the Institutional Animal Care and Use Committee (IACUC) of Novartis, Cambridge, MA, USA and following the guidance of the Association for Assessment and Accreditation of Laboratory Animal Care (AAALAC)

Note that full information on the approval of the study protocol must also be provided in the manuscript.
