# Supplementary figures and images for: Activation of human STING by a molecular glue-like compound
Source: Nat Chem Biol. 2023 Oct 12;20(3):365–72. doi: 10.1038/s41589-023-01434-y (PMC10907298; doi:10.1038/s41589-023-01434-y)

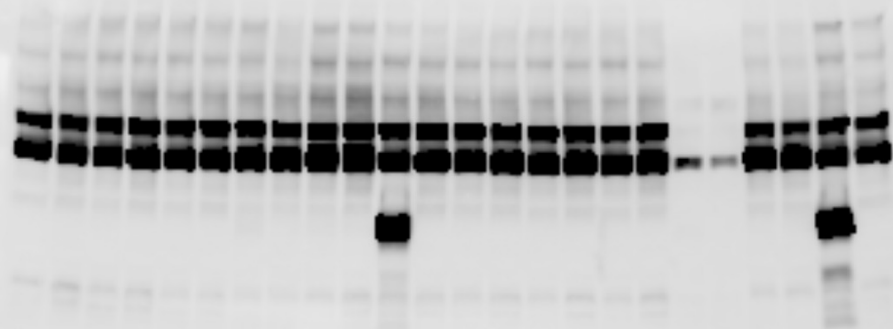

Supplement: Supplementary file 5 — Uncropped and unprocessed gels. [file 41589_2023_1434_MOESM5_ESM.pdf]

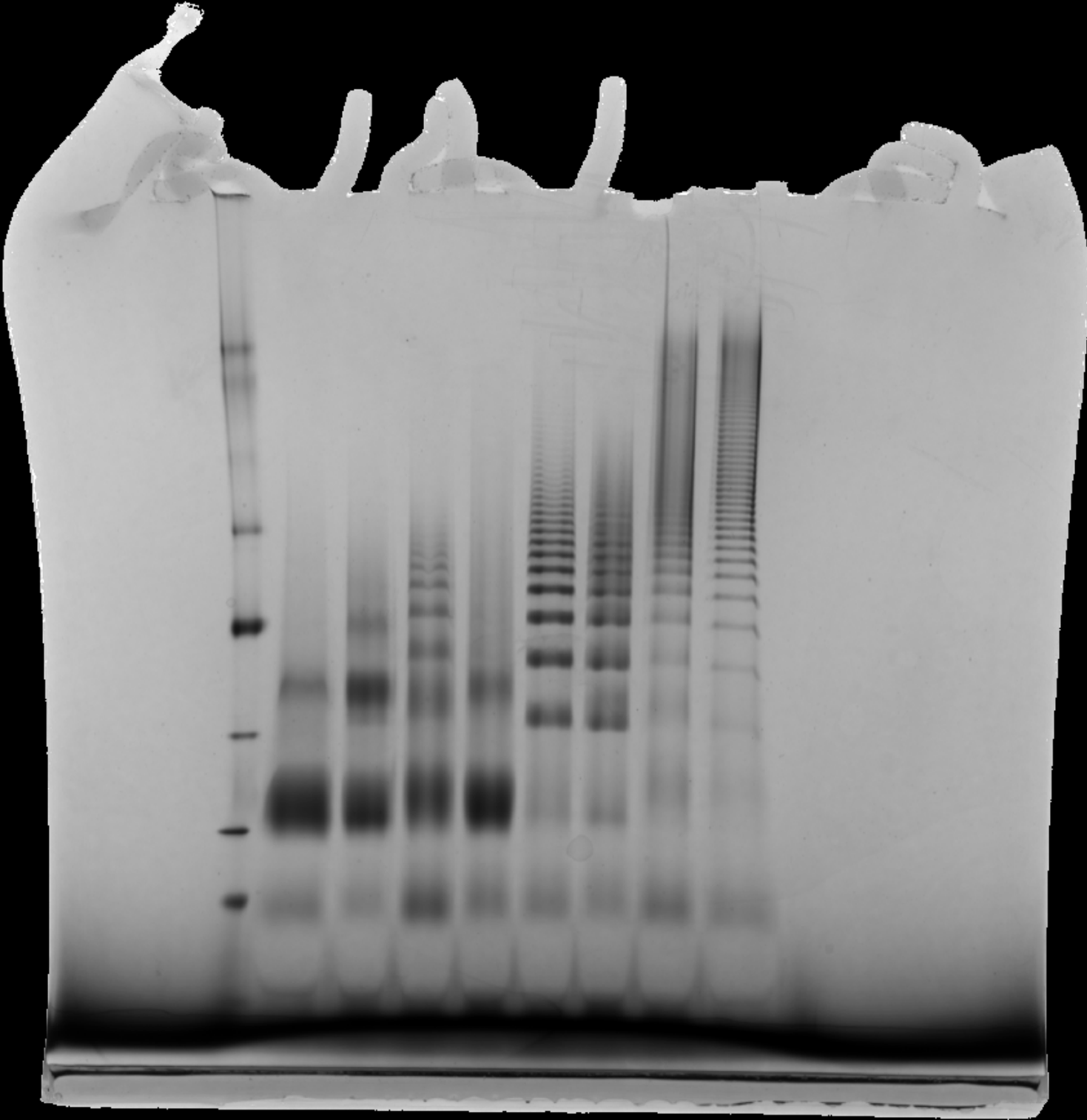

Supplement: Supplementary file 6 — Uncropped and unprocessed gels. [file 41589_2023_1434_MOESM6_ESM.pdf]
