# Supplementary material for: Activation of human STING by a molecular glue-like compound
Source: Nat Chem Biol. 2023 Oct 12;20(3):365–72. doi: 10.1038/s41589-023-01434-y (PMC10907298; doi:10.1038/s41589-023-01434-y)
Supplement: Supplementary file 10 — Uncropped and unprocessed gels and raw images. [file 41589_2023_1434_MOESM10_ESM.pdf]

Figure 6a

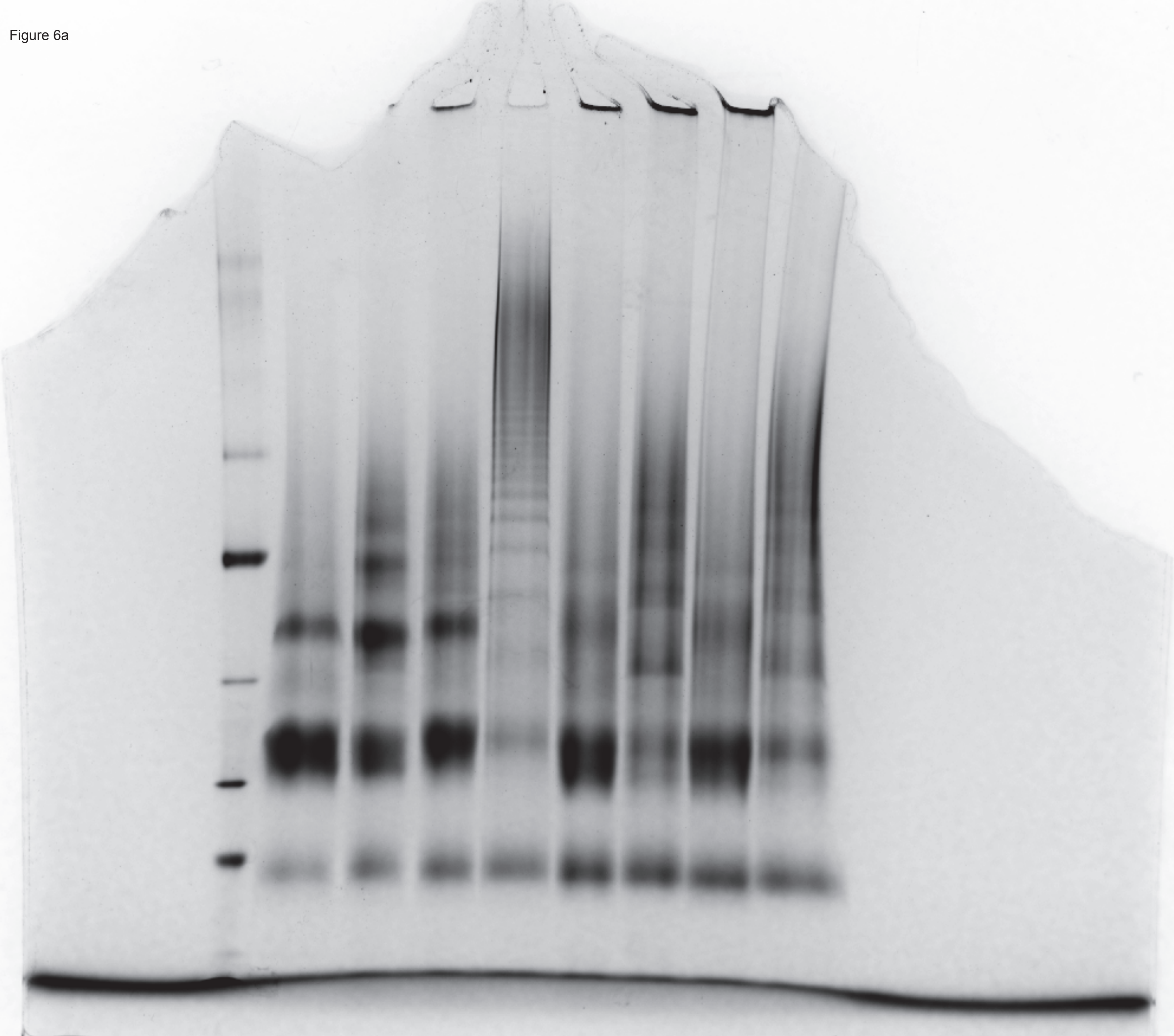

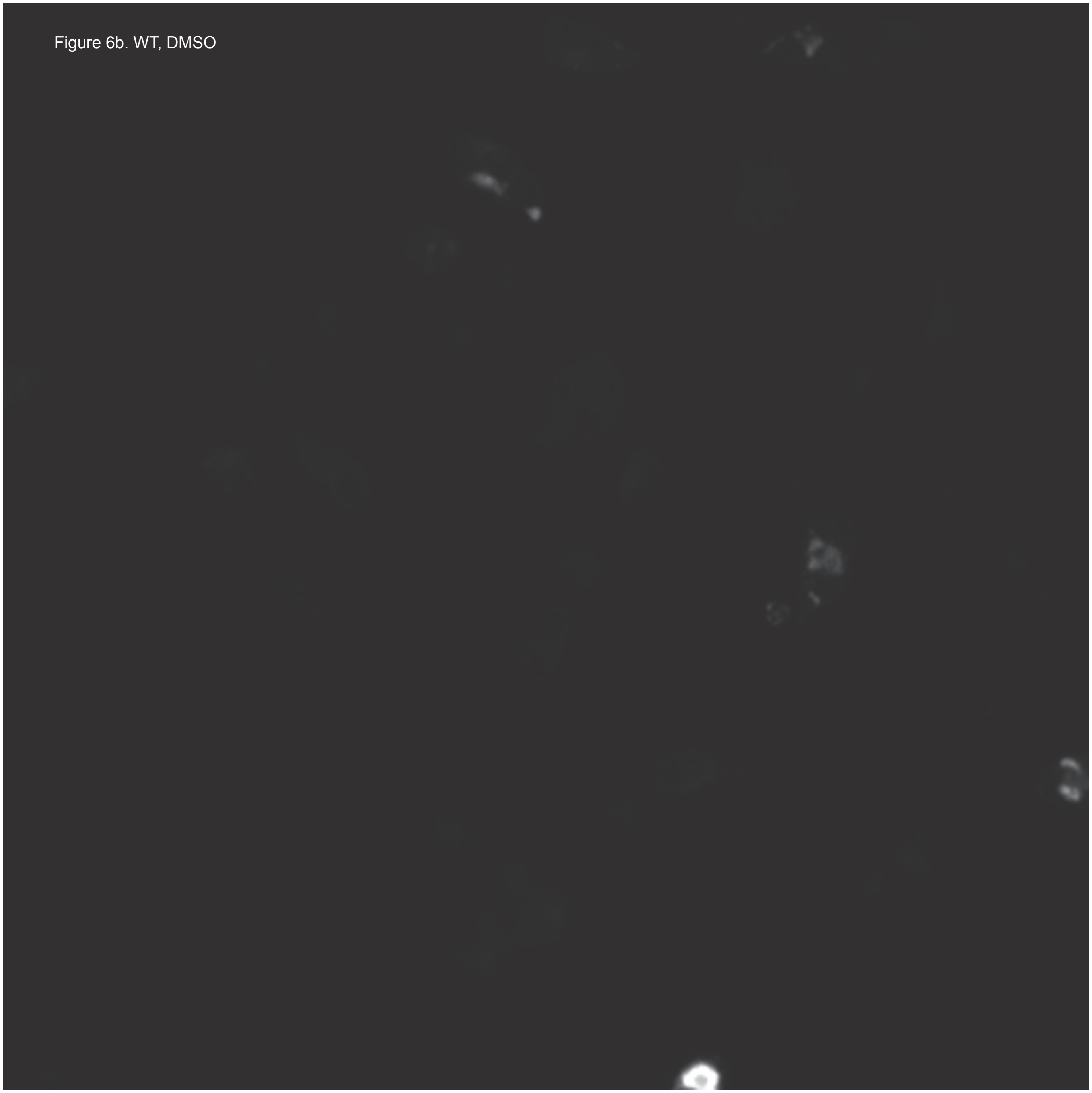
Figure 6b. WT, DMSO

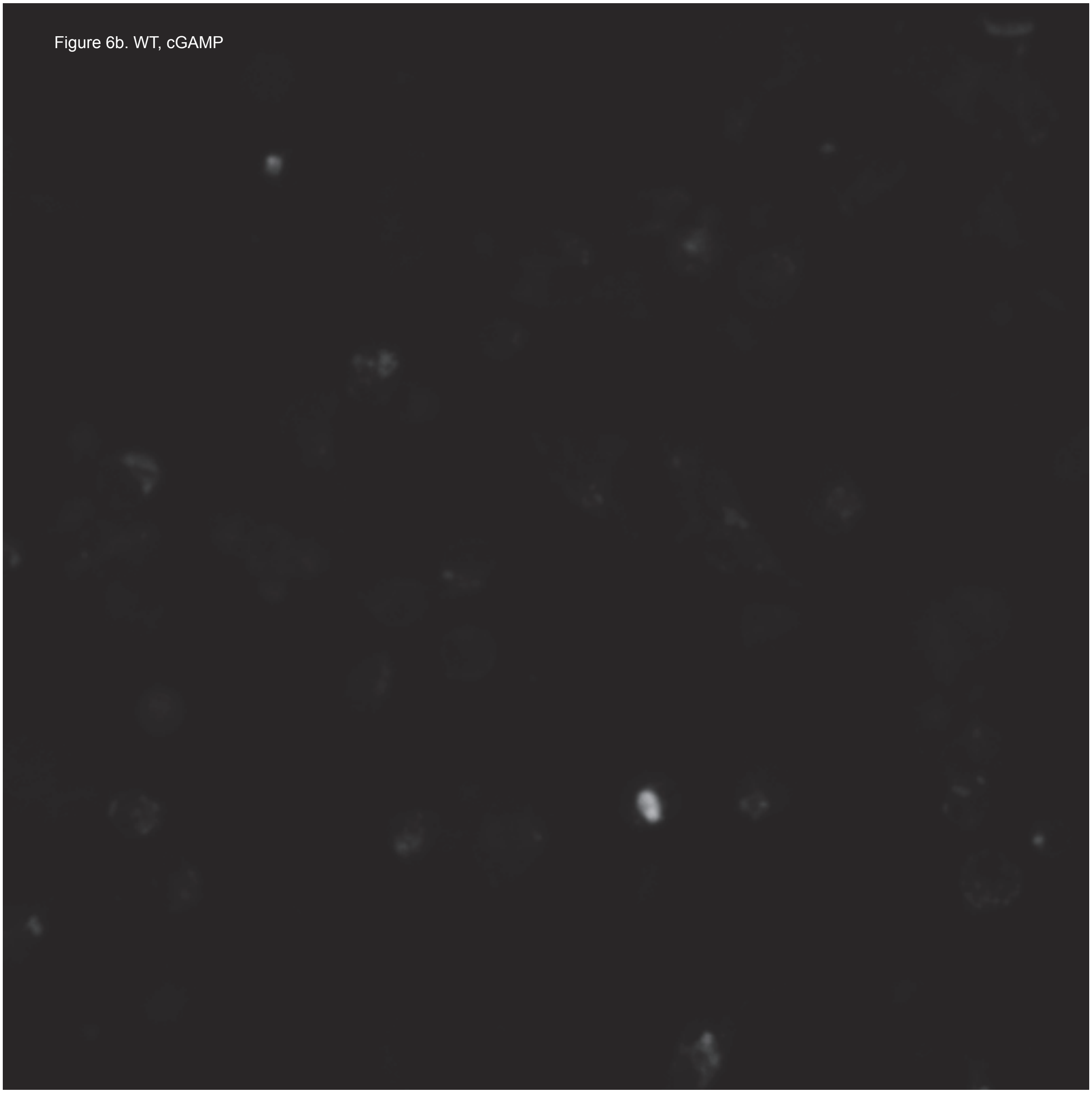
Figure 6b. WT, cGAMP

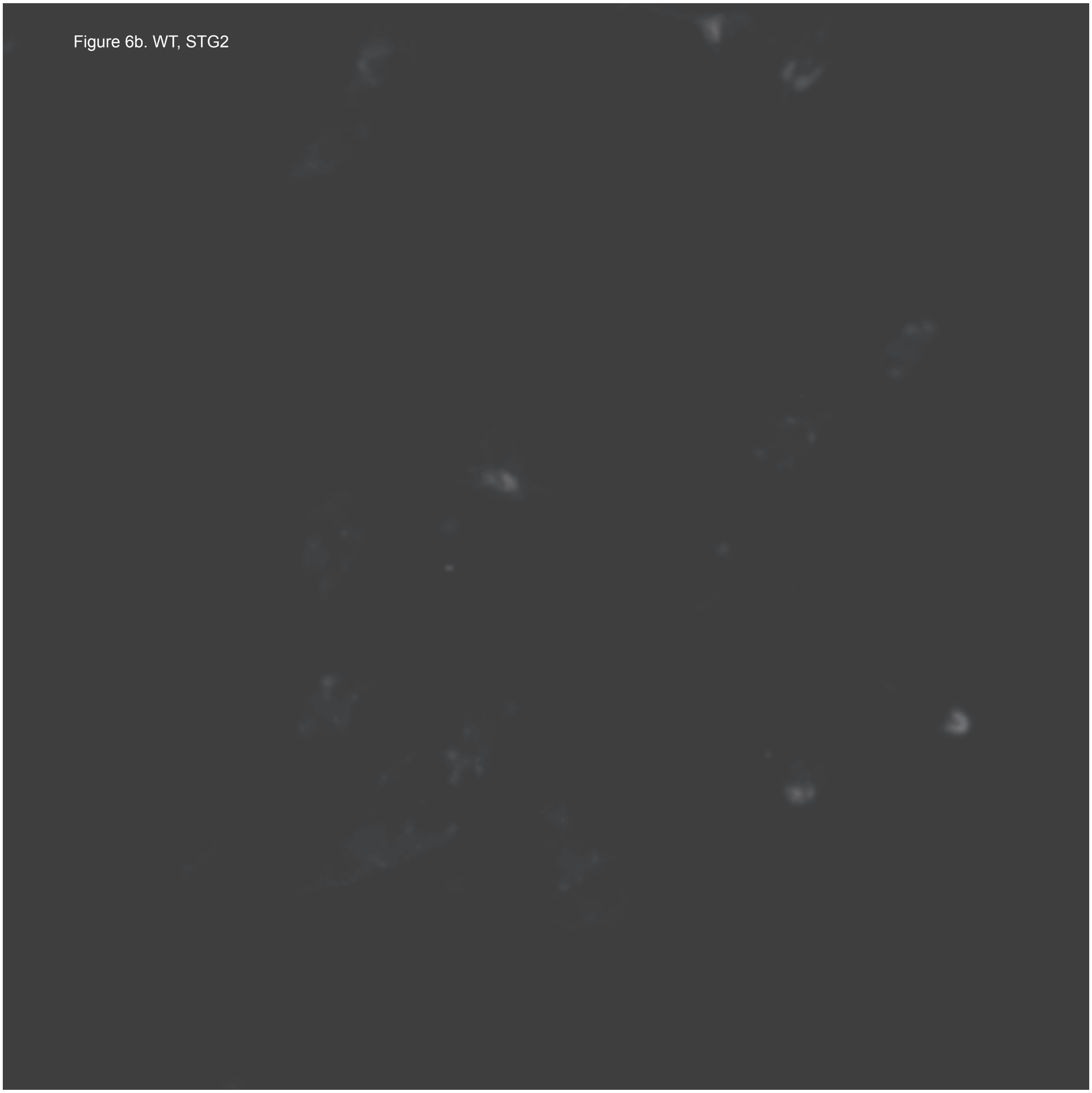
Figure 6b. WT, STG2

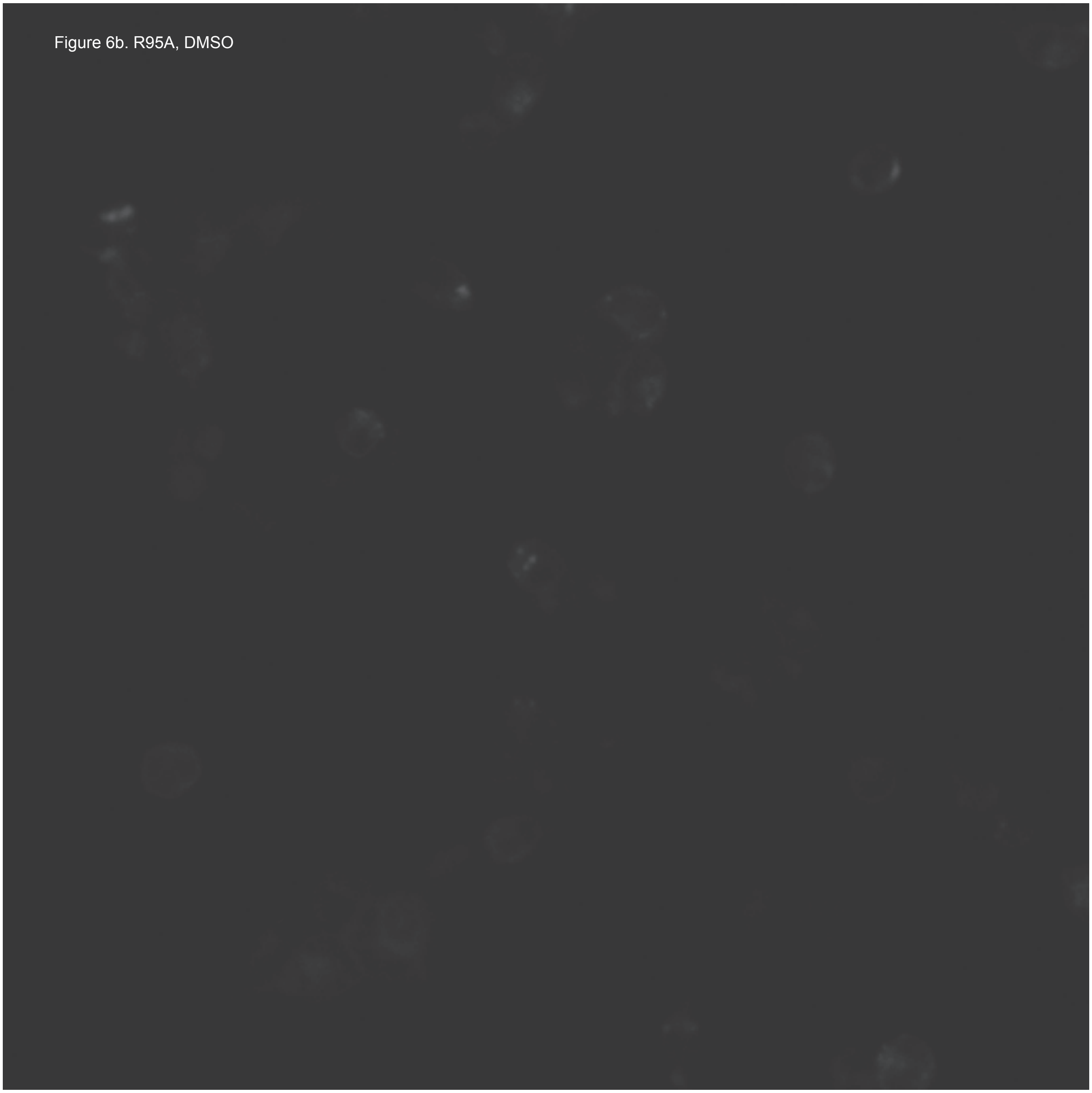
Figure 6b. R95A, DMSO

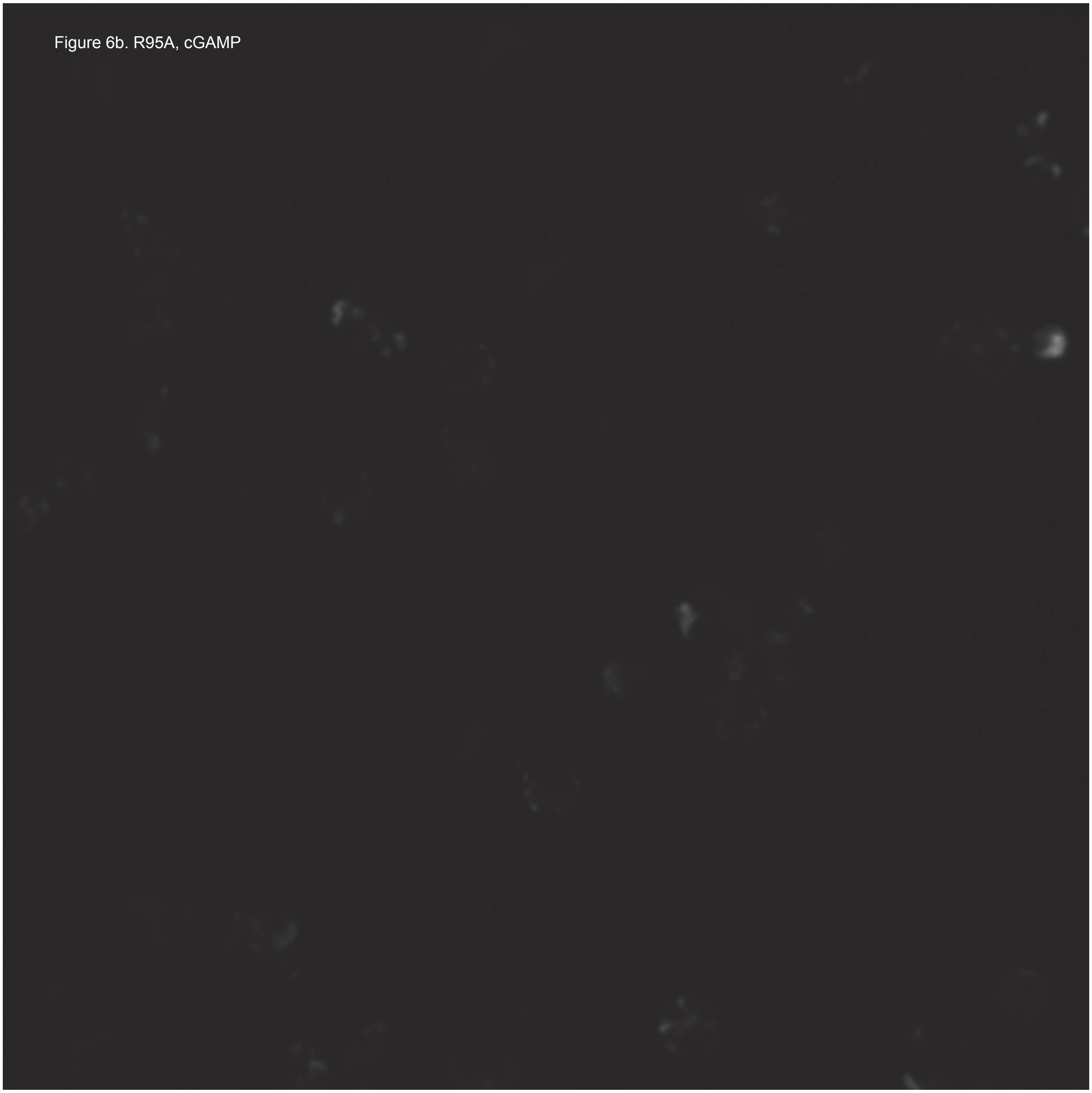
Figure 6b. R95A, cGAMP

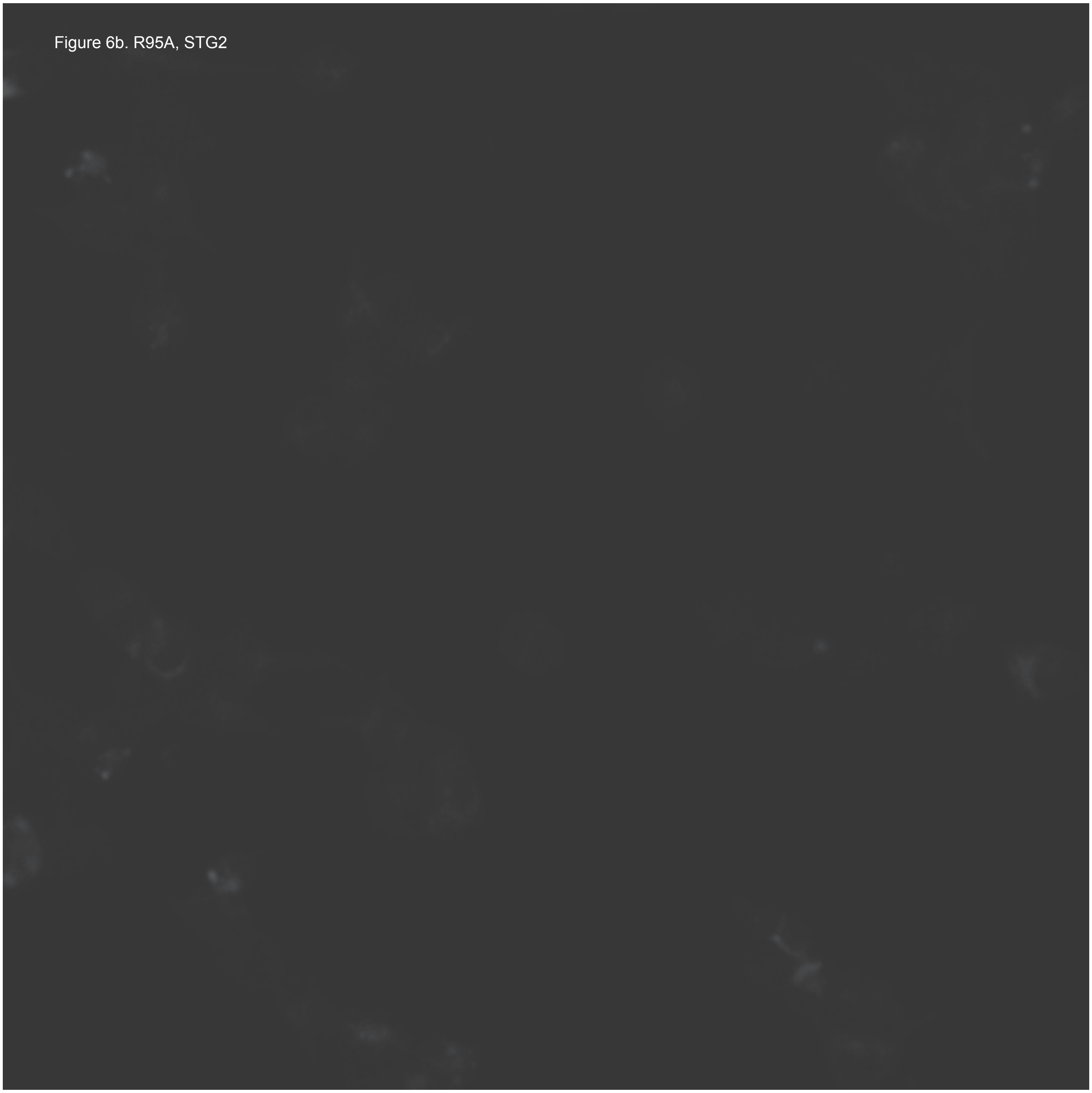

Figure 6b. R95A, STG2
